# Thyroid hormone receptor alpha sumoylation modulates white adipose tissue stores

**DOI:** 10.1038/s41598-021-03491-6

**Published:** 2021-12-16

**Authors:** Yan-Yun Liu, Jingjing Jiang, Sujie Ke, Anna Milanesi, Kiyomi Abe, Gilberto Gastelum, Jianrong Li, Gregory A. Brent

**Affiliations:** 1 Division of Endocrinology, Diabetes and Metabolism, Departments of Medicine and Physiology, David Geffen School of Medicine at UCLA, and Veterans Affairs Greater Los Angeles Healthcare System, Los Angeles, CA 90073 USA; 2 Present Address: Department of Endocrinology, Zhongshan Hospital, Fudan University, Shanghai, 200032 China; 3 Present Address: Department of Endocrinology, Union Hospital, Fujian Medical University, Fuzhou, 350001 China; 4 Present Address: Department of Pediatrics, Keio University School of Medicine, Tokyo, 160-8582 Japan; 5 Present Address: Saiseikai Central Hospital, Tokyo, 108-0073 Japan

**Keywords:** Cell biology, Molecular biology, Endocrine system and metabolic diseases

## Abstract

Thyroid hormone (TH) and thyroid hormone receptor (THR) regulate stem cell proliferation and differentiation during development, as well as during tissue renewal and repair in the adult. THR undergoes posttranslational modification by small ubiquitin-like modifier (SUMO). We generated the THRA (K283Q/K288R)^−/−^ mouse model for in vivo studies and used human primary preadipocytes expressing the THRA sumoylation mutant (K283R/K288R) and isolated preadipocytes from mutant mice for in vitro studies. THRA mutant mice had reduced white adipose stores and reduced adipocyte cell diameter on a chow diet, compared to wild-type, and these differences were further enhanced after a high fat diet. Reduced preadipocyte proliferation in mutant mice, compared to wt, was shown after in vivo labeling of preadipocytes with EdU and in preadipocytes isolated from mice fat stores and studied in vitro. Mice with the desumoylated THRA had disruptions in cell cycle G_1_/S transition and this was associated with a reduction in the availability of cyclin D2 and cyclin-dependent kinase 2. The genes coding for cyclin D1, cyclin D2, cyclin-dependent kinase 2 and Culin3 are stimulated by cAMP Response Element Binding Protein (CREB) and contain CREB Response Elements (CREs) in their regulatory regions. We demonstrate, by Chromatin Immunoprecipitation (ChIP) assay, that in mice with the THRA K283Q/K288R mutant there was reduced CREB binding to the CRE. Mice with a THRA sumoylation mutant had reduced fat stores on chow and high fat diets and reduced adipocyte diameter.

## Introduction

Thyroid hormone (TH) and its receptor (THR) play an important role in embryonic development by stimulating cell proliferation and differentiation. A range of models, including frog, mouse, and zebrafish, have shown key roles for TH/THR signaling in development of the brain, retina, inner ear, heart, intestine and bone^1^. In postnatal development and adulthood, THR/TH regulatory networks impact the balance of stem cell proliferation and differentiation, which are essential for tissue renewal and regeneration after injury^2,3^.

White adipose tissue (WAT) originates from embryonic mesoderm. Adipose precursor cells (preadipocytes) proliferate and differentiate to maintain adipose tissue homeostasis. The metabolic impact of WAT depots in humans varies by location, with upper-body subcutaneous and visceral fat associated with insulin resistance and greater risk of cardio-metabolic diseases and lower-body subcutaneous femoral and gluteal fat metabolically protective^4^. Clinical studies have shown the importance of preadipocytes in maintaining adipose tissue and adipocyte turnover is a characteristic of healthy adipose tissue. It has been reported that the fraction of preadipocytes in fat stores is reduced in obesity^5–7^. THRA/TH plays an important role in the regulation of adipose stores stimulating lipolysis and lipogenesis as well as mediating TH and adrenergic signaling in fat^8–12^.

Posttranslational modification of THR by SUMO directly alters THR interaction with transcription factors and modulates transcription independent of ligand^13^. THRA has two sumoylation sites at lysine 283 and lysine 389. An alternate sumoylation site is at lysine 288, if lysine 283 is blocked. We have previously shown that mutation of the sumoylation sites in THRA and THRB reduce gene expression of Wnt ligand and alter the canonical Wnt/β-catenin signaling pathway, which reduces adipocyte proliferation and adipogenesis in a T3-independent fashion^14^. An in vivo model of desumoylated THRB shows disruption of thyroid stimulating hormone (TSH) regulation in the pituitary and thyroid hormone synthesis in the thyroid gland, both triiodothyronine (T3)-independent^15^. Desumoylated THRB disrupted transcription factor binding to the TSH beta gene promoter and reduced cAMP-response element binding protein (CREB) binding to thyroid hormone synthesis genes.

In the present study, we investigated the in vivo role of THRA sumoylation*,* using THRA K283Q/K288R sumoylation mutant mice and human primary preadipocytes. Mice with the THRA K283Q/K288R mutation had reduced fat stores on chow and high fat diets, as well as reduced adipocyte cell diameter. Preadipocyte proliferation was reduced in human preadipocytes and in THRA K283Q/K288R mutant mice using in vivo and in vitro labeling approaches. Mice with the desumoylated THRA had disruptions in cell cycle G_1_/S transition and this was associated with a reduction in the availability of cyclin D2 and cyclin-dependent kinase 2.

## Results

**Figure 1:**
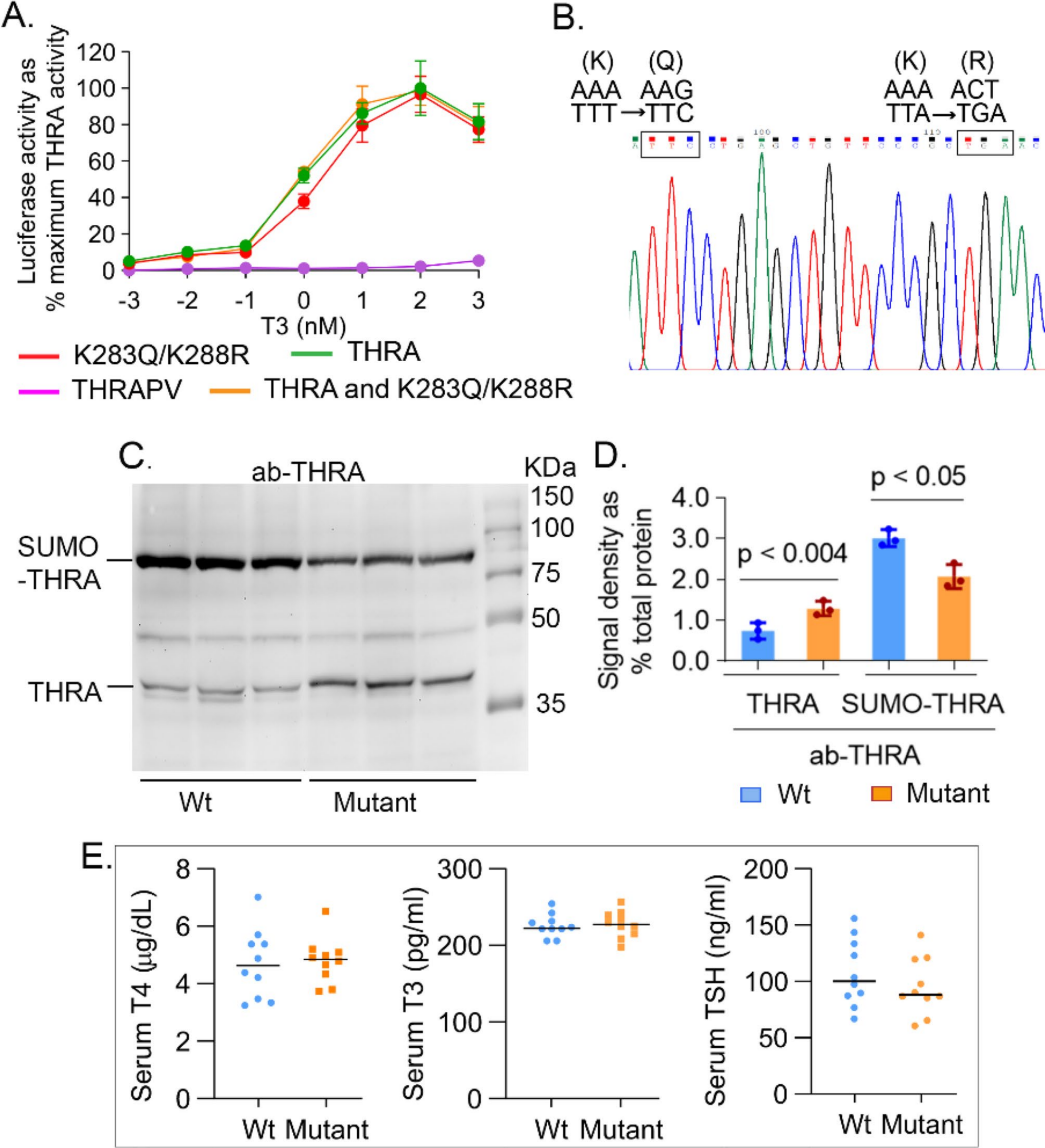
Genotype and thyroid function tests in THRA K283Q/K288R mutant mice. (**A**) A reporter assay was performed to test the T3 induction of the mutant THRA K283Q/K288R on T3-mediated luciferase reporter expression. JEG3 cells were transfected with THRA expression vectors and a luciferase reporter containing consensus 3 × Thyroid Hormone Response Elements. The THRA PV mutant receptor, based on mutations in Resistance to Thyroid Hormone, was used as a negative control since it does not bind to T3. The combination of THRA with THRA K283Q/K288R was used to determine dominant-negative effects of the THRA K283Q/K288R mutant receptor. (**B**) The presence of the mutation in homozygous THRA K283Q/K288R mutant mice was confirmed using direct DNA sequencing. (C-D) Western blot detection of THRA and sumoylated THRA, based on protein molecular weight. Subcutaneous fat was dissected from mice (n = 3/genotype). Protein lysate (35 μg/lane) was loaded on a 10% SDS gel. Antibody used was ab-THRA (**C**). (**D**) Quantification of THRA and sumoylated THRA in Western blot. The antibody detected bands and total protein (from Ponceau stained membrane, see Supplementary Fig. S2 online) were quantified using Li-Cor Image Studio lite. The data was normalized for Ponceau stained protein and presented as % total protein. Each bar in the figure represents the data from three mice. The significance was determined using Student *t*-test. (**E**) Fasting serum thyroxine (T4), triiodothyronine (T3) and Thyroid Stimulating Hormone (TSH) concentrations in Wt and THRA K283Q/K288R mutant mice, individual levels shown for Wild type (Wt) (circles) and mutant (squares), with a horizontal line showing the mean (n = 10). Statistical analysis was performed by Student *t*-test.

THRA has two sumoylation sites, K283 and K389. Lysine 389 interacts with the ligand binding cavity and mutations of this site diminish ligand binding. We mutated K283 to reduce THRA sumoylation without disrupting ligand binding. Mutation of the K283 residue, however, results in a shift of the SUMO conjugation site to K288^14^. We, therefore, generated THRA K283Q/K288R, a double mutation, to eliminate SUMO binding to this THRA domain, but retain ligand binding. The double mutation, THRA K283Q/K288R, was assessed in a functional assay with a consensus 3 × Direct Repeat (DR) 4-Thyroid Hormone Response Element (TRE)-Luciferase (Luc) reporter. The magnitude of T3-induction with THRA K283Q/K288R was equal to THRA alone, or a combination of wild type and mutant THRA (Fig. 1A).

### Metabolic characterization of THRA K283Q/K288R mutant mice

**Figure 2:**
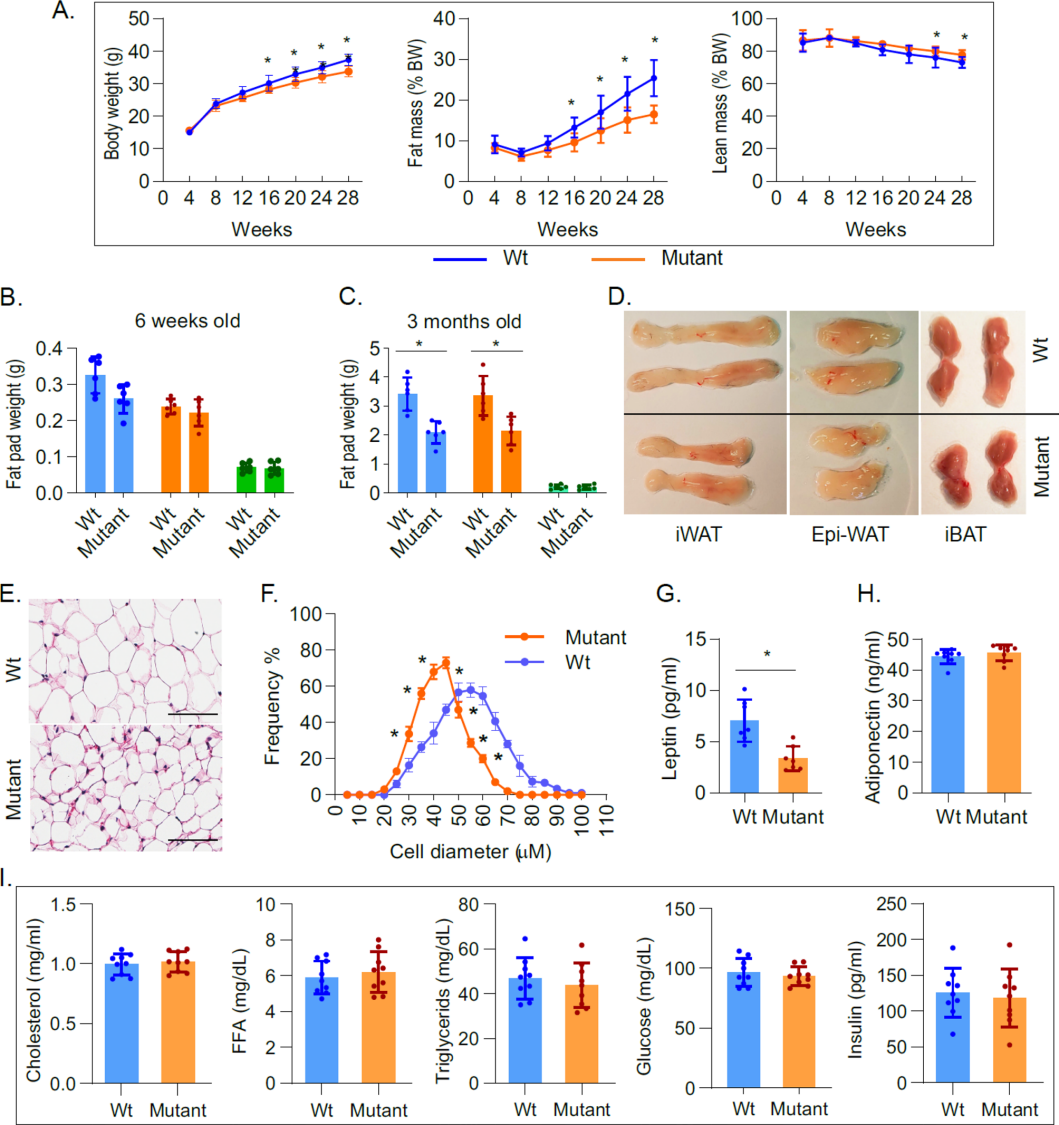
THRA K283Q/K288R mutant mice display body composition, fat stores, and metabolic profile. Age-matched wild type (Wt) and THRA K283Q/K288R mutant mice (mutant) were fed with chow diet. (**A**) Body composition was determined by Echo-MRI (n = 9/group) and average value is shown for body weight (BW), fat and lean body mass. (**B**) Fat pads (iWAT-inguinal White Adipose Tissue-blue bars, Epi- epididymal fat-orange bars, iBAT- inter-scapular Brown Adipose Tissue-green bars) were dissected and weighed from 6 weeks old mice (n = 6) and from (**C**) 3 month old mice (n = 6). (**D**) Images of dissected fat pads from 3 month old Wt and mutant mice (n = 6/group), representative samples are shown. (**E**) Histological analysis of inguinal WAT, representative slides from wild type and mutant mice are shown (scale bar 100 μm). (**F**) Fat cell diameter (μM) of inguinal fat was analyzed. A total of 360 cells from each genotype was quantified (see “Materials and methods” section for details). The data is shown as diameter frequency of 360 cells. (**G**,**H**) Non-fasting serum leptin and adiponectin levels (n = 7/group, 3 month old mice). (**I**) Fasting serum cholesterol, triglycerides, free fatty acids (FFA), insulin and glucose concentrations in 3 month old mice. The statistical analysis was performed using paired Student *t*-test.

THRA K283Q/K288R mice were generated as a global mutation (see Supplementary Fig. S1 online) and homozygosity was confirmed by sequencing (Fig. 1B). We determined the impact of the introduced THRA mutations on the extent of THRA sumoylation in subcutaneous adipose tissue, based on the size of the band on a Western blot probed with ab-THRA. We found that the desumoylated THRA was increased 73% (SE ± 14%, *P* < 0.04) and the sumoylated THRA reduced 34% (SE ± 13%, *P* < 0.05), in adipose tissue from the mutant, compared to Wt, mice (Fig. 1C,D and see Supplementary Fig. S2 online). These data indicate that the sumoylation site mutations significantly reduced sumoylated THRA and increased desumoylated THRA in adipose tissue in vivo*,* compared to Wt mice. The amount of total THRA protein, the sum of sumoylated and desumoylated THRA, was not different in THRA mutant compared to Wt mice (3.7% of total protein in control and 3.4% in mutant, P < 0.35). The total protein was calculated based on the amount detected by Ponceau red stain (Fig. S2). T3-dependent gene expression in adipose tissue was assessed by RNAseq and there was no significant difference in the expression of T3-responsive genes in inguinal fat in the mutant mice, compared to Wt (see Supplementary Fig. S3 online). These data indicate that are mutation strategy successfully reduced THRA sumoylation, but preserved classic T3 signaling in vivo. The serum thyroxine (T4) (Wt mice, 4.71 μg/dL ± SD 1.21; mutant mice 4.82 μg/dL ± SD 0.83), serum triiodothyronine (T3) (Wt 218 pg/mL ± SD 22; Mutant 234 pg/mL ± SD 49.6), and Thyroid Stimulating Hormone (TSH) (Wt 107.8 ng/mL ± SD 28.3; Mutant 95.5 ng/mL  ± SD 25.2), were not significantly different in mutant mice compared to Wt mice (Fig. 1E). The body weight of the mutant mice was significantly lower, compared to Wt mice, beginning at age 20 weeks (p < 0.037) (Fig. 2A). Body composition, as determined by Echo-MRI, showed that the percent body fat of the mutant mice was significantly reduced, compared to Wt mice, from age 16 weeks onward (Fig. 2A). The inguinal White Adipose Tissue (iWAT) and epididymal (Epi-WAT) fat pads from 6 week-old mice were not different in weight from mutant mice (Fig. 2B), however, the iWAT and Epi-WAT fat pads from 3 month old mutant mice were reduced 39% (p < 0.002) and 35% (p < 0.004) respectively, compared to Wt mice (Fig. 2C). The inter-scapular brown adipose tissue (iBAT) in THRA K283Q/K288R mice was not significantly different from Wt, in juveniles (6 week) or adult mice (3 months), indicating that THRA sumoylation impacted WAT, but not BAT stores. The dissected fat pads from iWAT and Epi-WAT, were smaller in size in the mutant mice compared with Wt, consistent with overall reduced body fat (Fig. 2D). Histological analysis of iWAT showed that the adipocytes in mutant mice had a smaller diameter than Wt (Fig. 2E). The adipocyte diameter was quantified from 3 mice of each genotype and the fat cell diameter in iWAT from mutant mice was significantly smaller than that from Wt mice (Fig. 2F). The serum leptin of the mutant mice was reduced to 52% of the level in Wt mice (p < 0.014) (Fig. 2G), consistent with overall reduced body fat. The serum adiponectin level was not different between Wt and mutant mice (Fig. 2H). There was no difference in fasting serum cholesterol, free fatty acid, triglycerides or insulin concentrations, comparing the mutant mice fed with chow diet at 3 months of age to Wt mice (Fig. 2I). Food intake was not significantly different in mutant compared to Wt mice (see Supplementary Fig. S4 online). The adipose tissue phenotype in the mutant mice demonstrated a distinct role of THRA sumoylation in regulating white adipocyte diameter and the size of WAT body stores^14^.

### Influence of high fat diet on body weight and fat stores

**Figure 3:**
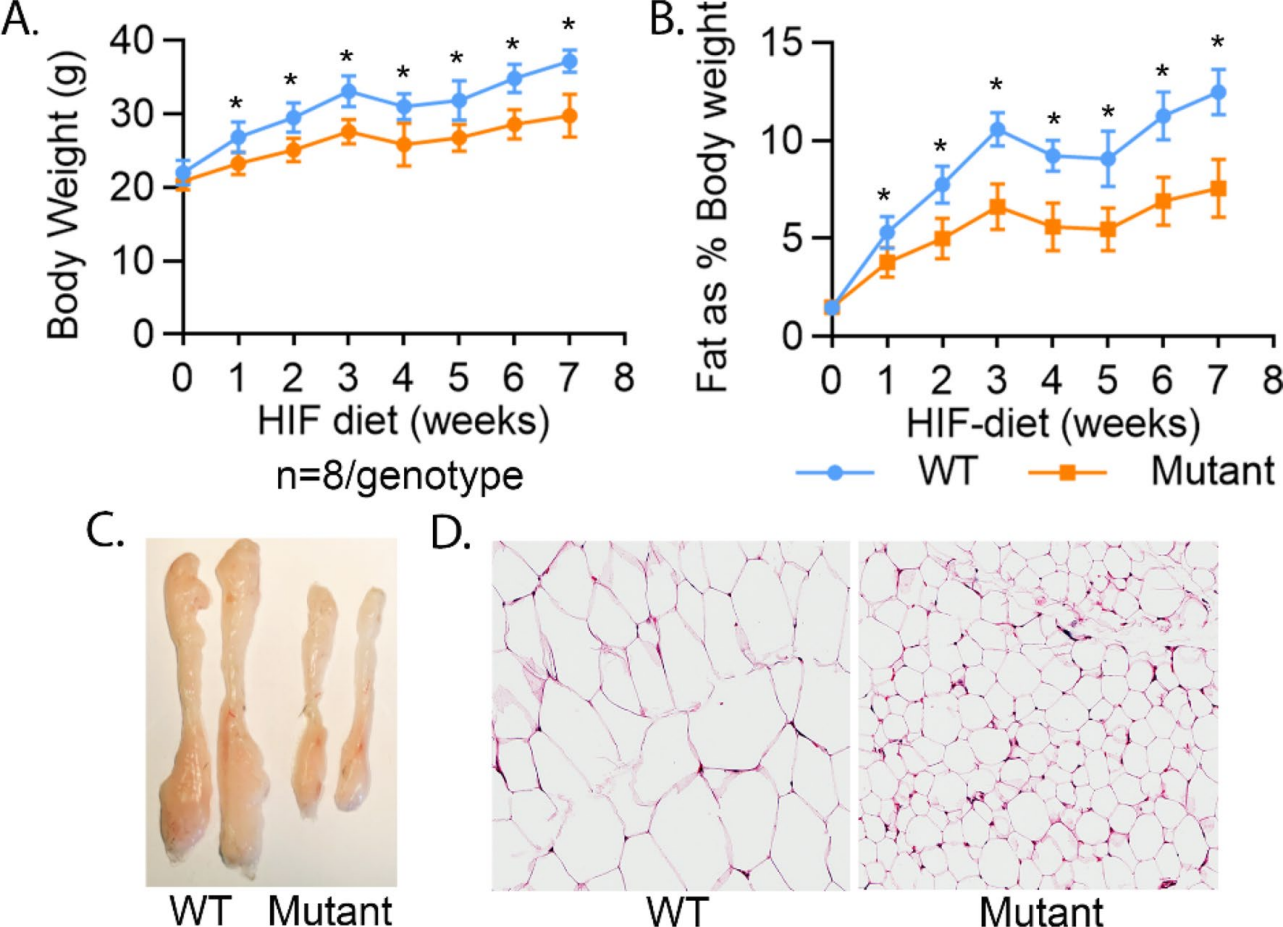
THRA K283Q/K288R mice are resistant to High fat diet-induced body fat increase and adipose tissue expansion. Mice (n = 8/genotype) were given High Fat (HIF) diet (40% fat in calories) for 7 weeks starting at age 7 weeks old. The body composition was measured by Eco-MRI. (**A**) Body weight and (**B**) body fat as % body weight. (**C**) Mice were euthanized and fat pads were dissected, rinsed and photographed and representative inguinal fat depot as shown. (**D**) Representative histological image of inguinal fat from WT and mutant mice. The statistical analysis was perform at each time point using paired Student *t*-test.

To determine the effect of a high fat diet, we fed 7 week old mutant and wild-type mice a Western diet (40% of calories from fat) for 7 weeks. The body composition was measured by Eco-MRI. The mutant mice gained markedly less weight and had lower per cent body fat, compared to wild-type mouse (Fig. 3A,B). Inguinal fat stores were substantially reduced in mutant, compared to Wt mice (Fig. 3C) and the fat cell diameter was also substantially reduced in the mutant mice compared to Wt (Fig. 3D). Food intake was not significantly different between wild type and mutant mice (mean 5.88 ± SD 1.79 g/day/mouse vs 6.33 ± SD 1.80 g/day/mouse, p = 0.58). The difference in fat stores in mutant mice compared to Wt mice, after a high fat diet, were in the same direction as those seen on a chow diet, but the difference between mutant and Wt mice was enhanced.

### Preadipocytes proliferation in mutant mice

**Figure 4:**
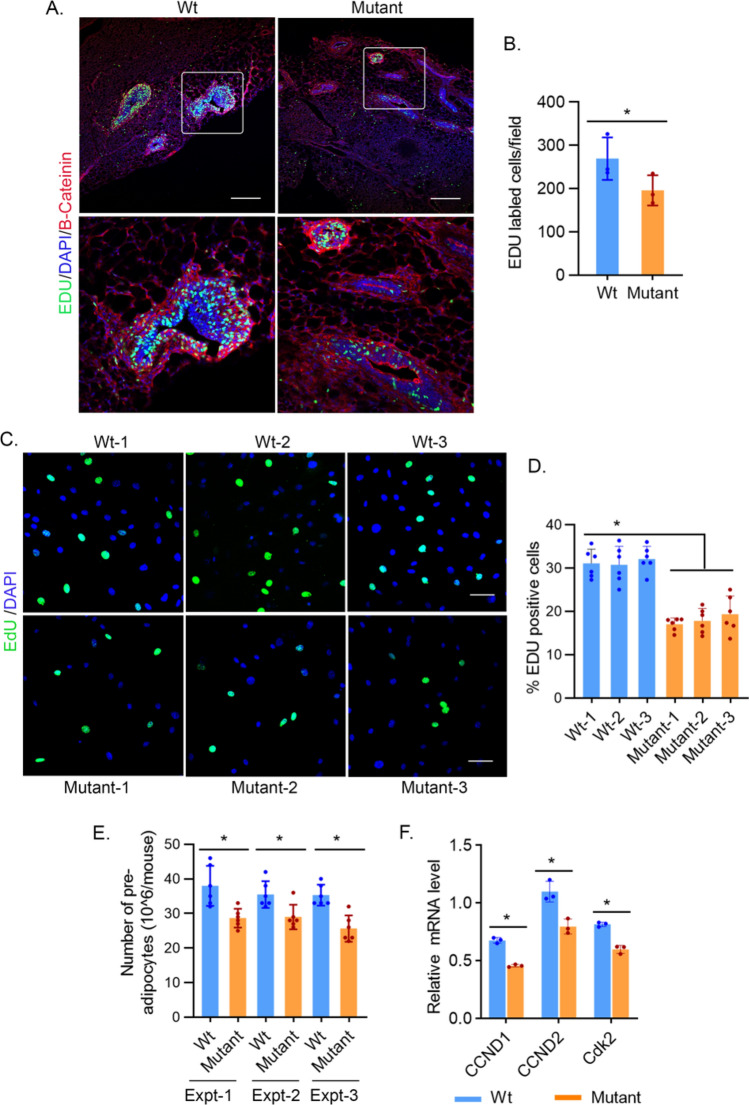
Preadipocyte proliferation in mutant mice determined in vivo and in vitro. (**A**,**B**) EdU labeling of proliferating preadipocytes in 3 week old mice. Mice were given EdU, 5 μg/100 g body weight (i.p.) and inguinal fat pads were isolated and fixed in 4% paraformaldehyde 12 h after injection. Frozen sections (12 μM thickness) of inguinal fat depots were stained with β-catenin (red) for cell membrane and DAPI (blue) for nuclei. EdU (Green) incorporation of DNA in dividing cells was detected by Click-IT chemistry. (**A**) EdU incorporation was imaged using confocal microscope and representative sections of regions with stromal vascular regions are shown. Lower panel shows enlarged view of the area as indicated. The scale bar is 200 μM. (**B**) EDU incorporation was quantified in three different sections using a × 20 magnification field. (**C**) Isolated preadipocytes proliferation. Preadipocytes were derived from the stromal vascular fraction of fat from 6 week old wild type (Wt) and mutant mice (n = 6/group). Preadipocytes from each mouse were plated on separate plates and synchronized as described in the “Materials and methods” section. After synchronization, cells were supplemented with 10% serum and labeled with EDU for 4 h, followed by fixation and imaging. Representative images from 3 separate experiments are shown. Scale bar is 50 μM. (**D**) Quantification of EDU positive cells (green color) and nuclei imaged with DAPI (blue). (**E**) Quantification of the number of SVF-derived preadipocytes. For each experiment (n = 6 mice). The data shown is the average numbers of preadipocytes in each experiment. (**F**) q-PCR analysis of Cyclin D1 (*CCND1)*, Cyclin D2 (C*CND2)* and cyclin-dependent kinase 2 (*Cdk2)* mRNA levels, obtained from expression PCR array and normalized for four housekeeping genes. Please see “Materials and methods” for quantification shown in (**B**) and (**C**). *, p < 0.05 compared to wild type mice.

We investigated whether the reduced fat mass in THRA K283Q/K288R mutant mice was associated with reduced preadipocyte proliferation. To determine the preadipocyte proliferation status in live mice, we performed EdU labeling of 3 week old mice. The EdU labeled-nuclei represent cells in S phase, a phase of active DNA synthesis. In adipose tissue, dividing preadipocytes are concentrated in the vascular stroma. The EdU labeled-nuclei were reduced 27% ± SD 5.2% (*p* < 0.02) in the vascular stroma of inguinal adipose tissue in THRA mutant mice, compared to Wt mice (Fig. 4A,B). We also isolated preadipocytes from the stromal vascular fraction of iWAT from 6 week old Wt and THRA mutant mice. Cells were synchronized to G_0_/G_1_ cell cycle by serum withdrawal for 36 h and then stimulated to enter the cell cycle by addition of a 10% serum supplement. The proliferation of preadipocytes isolated from THRA K283Q/K288R mutant mice, as determined by EdU-DNA incorporation, was reduced 24.3% ± SD 3.7% (*p* < 0.011) in mutant mice compared to Wt mice (Fig. 4C,D). The number of preadipocytes isolated from SVF of the iWAT of the mutant mice was reduced 23.3% ± SD 3.3% (p < 0.0035) from 3 individual experiments (n = 6 mice/experiment) compared to that of Wt mice (Fig. 4E). The mRNA levels of *Cyclin D1 and D2* and cyclin-dependent kinase 2 (*Cdk*2) were reduced in the mutant preadipocytes, compared to Wt mice (Fig. 4F).

### THRA sumoylation-defective mutant K283Q/K288R reduces nuclear localization of cyclin D1, cyclin D2, Cdk4 and Cdk2 in human primary preadipocytes

**Figure 5:**
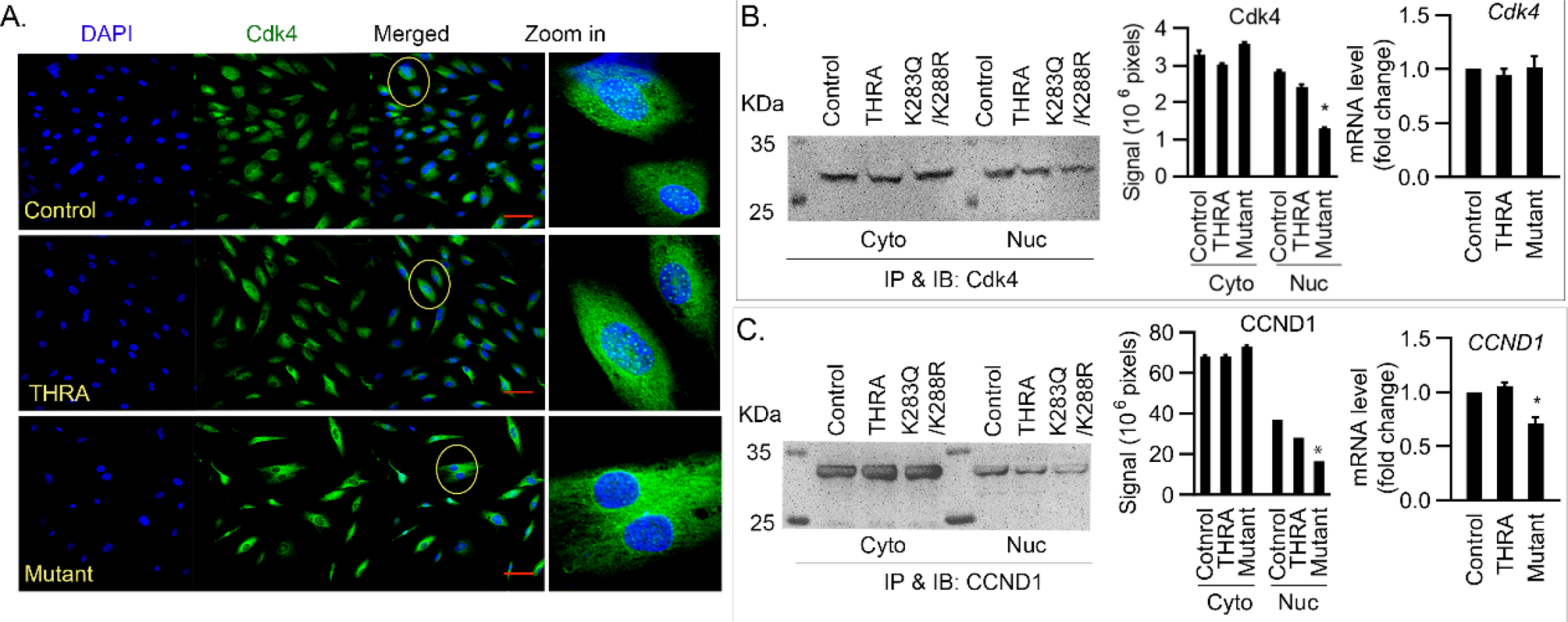
Mutation of the THRA sumoylation site, K283Q/K288R, associated with reduced nuclear localization of cyclin D1-Cdk4 complex, cyclin D2 and Cdk2 in G_1_ phase. (**A**) Human preadipocytes were transfected with empty vector (control) or plasmids expressing THRA or THRA K283Q/K288R. Cells were synchronized to G_0_/G_1_ phase by serum starvation for 48 h. Cells were then allowed to re-enter the cell cycle by supplementing the medium with 10% calf serum. After 9 h of serum supplementation, the cells were analyzed for nuclear content of cyclin D1-Cdk4 complex, cyclin D2 and Cdk2. Immunofluorescent (IF) staining of cyclin D1 and Cdk4 nuclear localization in cells. Scale bar, 50 μm. Circled inset shows higher magnification. (**B**) The nuclear and cytoplasmic fractions were analyzed for Cdk4 protein expression by IP with anti-Cdk4 Ab, followed by immunoblot (IB) with anti-Cdk4 and quantified Cdk4 IB signals using Li-Cor Image Studio Lite. (**C**) Co-IP detection of cyclin D1 in the cytoplasm and nuclear fractions. Anti-p27kip antibody was used in IP and anti-cyclin D1 antibody in IB. Quantification of WB was done using Image Studio Lite. q-PCR quantification of *Cdk4* and *cyclin D1 (CCND1)* mRNA expression, presented as the mean value and SD, fold above control. *p < 0.05 compare to control by One-way Anova. IP-immunoprecipitation, IB-immunoblot. The blots (**B**,**C**) were cut prior to hybridization and the origin blots are provided (see Supplementary Fig. S7 online).

The influence of the sumoylation defective THRA on proliferation was studied in human primary preadipocytes cells, transfected with plasmids expressing THRA, THRA K283Q/K288R, or an empty vector (control), synchronized to G_0_ by serum withdrawal for 48 h. Serum withdrawal resulted in more than 75% of the cells synchronized to G_0_ phase, as assessed by flow cytometry. Serum withdrawal prolonged the G_1_ and S_1_ phases, compared to growth in serum conditions, due to recovery from the lower cellular metabolism associated with serum starvation^16^. Cells were, therefore, supplemented with serum for 9 h and analyzed for the expression of G_1_ phase regulators. In the G_1_ phase, cyclin D1 forms a complex with cyclin-dependent kinase 4 (Cdk4) The cyclin D1/Cdk4 complex is then transported from the cytoplasm to the nucleus by p27kip Cdk inhibitor^17^. In cells transfected with THRA K283Q/K288R, compared to those transfected with empty vector (control) and THRA, the immunofluorescent (IF) staining demonstrated that Cdk4 protein was predominantly in the cytoplasm but not the nucleus (Fig. 5A). The cell lysate was fractionated into cytoplasmic and nuclear fractions and immunoprecipitated (IP) with antibodies against Cdk4 in order to quantitate the subcellular distribution of Cdk4. The quality of fractionation was confirmed by alpha tubulin staining for the cytoplasmic fraction and lamininB1 for the nuclear fraction (see Supplementary Fig. S5 online). In the cytoplasmic fraction, Cdk4 was similar among different samples. In the nuclear fractions, Cdk4 was reduced in cells expressing THRA K283Q/K288R, compared to control and THRA-transfected cells (Fig. 5B). Cdk4 enters the nucleus in the form of a cyclin D1/Cdk4 complex, carried by p27^kip^, so the reduced nuclear Cdk4 could be the result of reduced nuclear cyclin D1. To investigate this possibility, we performed a co-IP with anti-p27^kip^ and WB with anti-cyclin D1. We found that in the cells transfected with THRA K283Q/K288R, p27^kip^-bound nuclear cyclin D1 was only 45% of the control levels (Fig. 5C). *Cyclin D1* mRNA was reduced 28% (p < 0.033) but *CDk4 mRNA* was not changed (Fig. 5B,C far right panels), indicating that reduction of the cyclin D1/Cdk4 complex is associated with reduced cyclin D1 gene expression.

**Figure 6:**
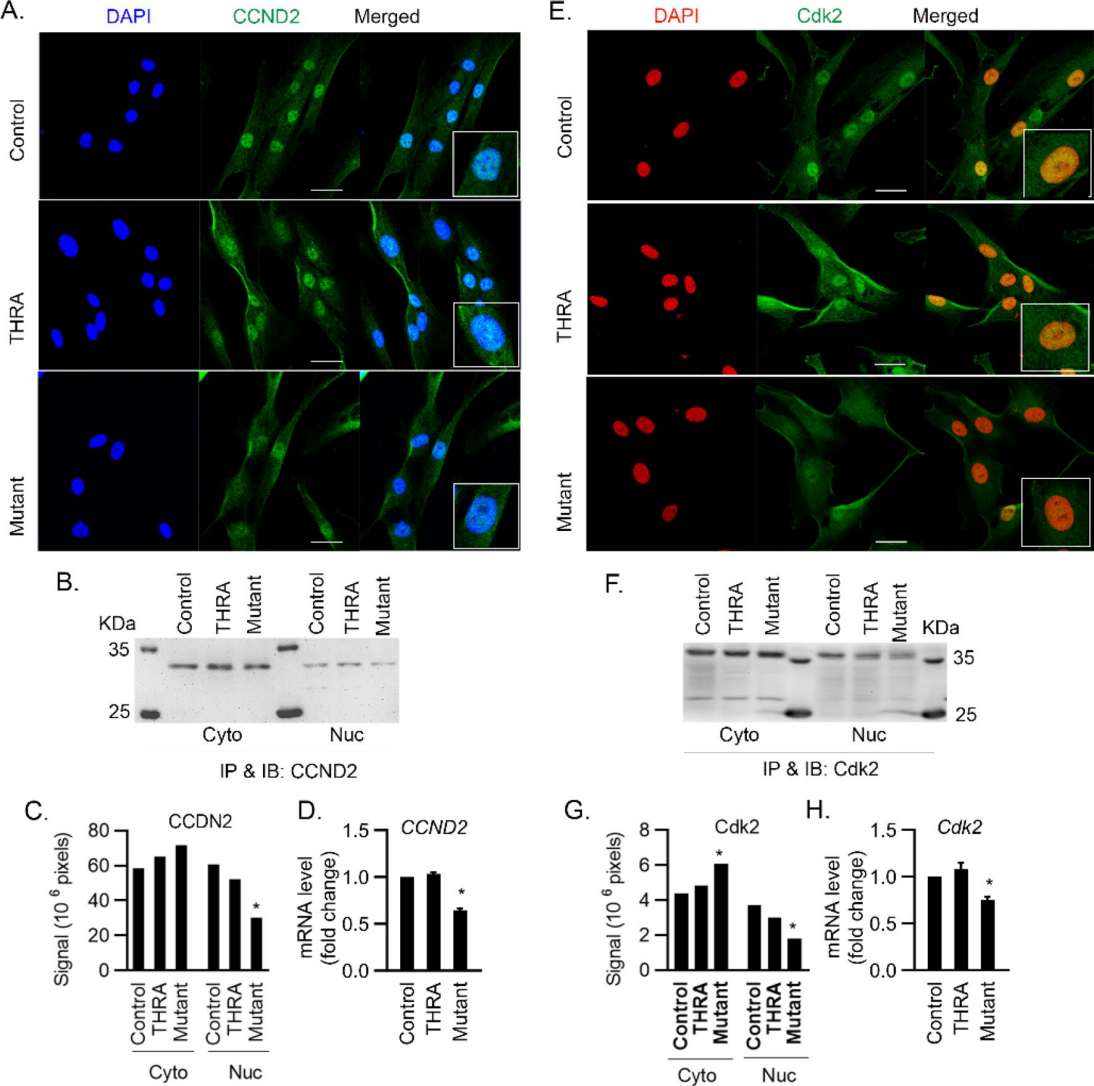
Nuclear localization of Cyclin D2 and Cdk2 in human primary preadipocytes expressing THRA K283Q/K288R. Cell treatment and conditions are the same as described in the Fig. 4 legend. (**A**,**E**) IF detection of nuclear cyclin D2 (CCND2) and Cdk2 protein with DAPI stain of nucleus. Circled inset shows higher magnification. (**B**,**F**) detection of nuclear CCND2 and Cdk2 protein using IP and followed by IB with anti-cyclin D2 antibody and anti-Cdk2 antibody, and quantified by Li-CoR Imaging Studio lite (**C**,**G**). (**D**,**H**) q-PCR analysis of *CCND2* and *Cdk2* mRNA and fold-change from control is shown. Primers were pre-designed (Qiagen). *p < 0.05 as statistically significant by One-way Anova for qPCR data, and by Two-way Anova for IB quantification Scale bar, 50 μm. The blots (panels B and F) were cut prior to hybridization and the origin blots are provided (see Supplementary Fig. S8 online).

Cyclin D2 is a G_1_/S phase-specific cyclin and forms a complex with Cdk4/6 that mediates the G_1_/S transition. We detected reduced nuclear cyclin D2 protein by immunofluorescence and IP of cytoplasmic and nuclear fractions in THRA K283Q/K288R-transfected cells, compared to THRA and control (Fig. 6A–C). *Cyclin D2* mRNA was decreased 36% in mutant cells compared to control (p < 0.034) (Fig. 6D). Cdk2 is another important factor regulating the G_1_/S phase transition. Cdk2 moves into the nucleus and, together with Cdk4, sequentially phosphorylates retinoblastoma protein (Rb). Cdk2, as shown by immunofluorescence staining and IP, localized to the nucleus in control and THRA-transfected cells. In THRA K283Q/K288R-tranfected cells, however, there was significantly less Cdk2 protein in the nucleus (Fig. 6E–G). Cdk2 mRNA levels were reduced 20% in THRA K283Q/K288R-transfected cells (Fig. 6H), indicating that reduced nuclear Cdk2 protein was associated with impaired Cdk2 gene expression and reduced nuclear translocation. These data show that expression of THRA K283Q/K288R is associated with slowing of the G_1_/S transition. We observed reduced nuclear localization of the cyclinD-Cdk complex and reduced mRNA levels of the cell cycle regulating genes, Cyclin D1, Cyclin D2 and Cdk2 in the G1 phase.

### Delayed G_1_/S transition

**Figure 7:**
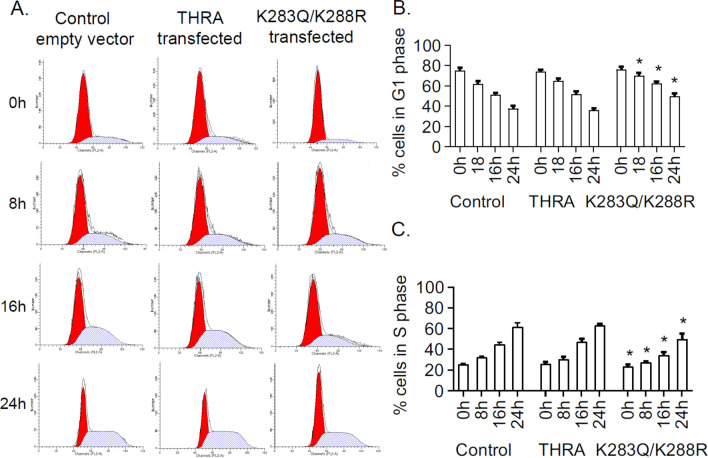
Analysis of cell cycle progression in human preadipocytes. (**A**) Cell cycle progression was analyzed by Propidium iodide Flow Cytometer. Cell synchronization is the same as describe in the Fig. 4 legend. After serum starvation, cells were supplemented with 10% serum for a period of 0, 8, 16, 24 h. At each time point, cells were collected and analyzed by flow cytometry. Percentage of cells in G_1_ phase (**B**) and S phase (**C**), obtained from flow cytometry for each time point is shown. *P < 0.05 as statistically significant compared with control and THRA at the same time point by One-way Anova analysis.

We performed cell cycle analysis by Flow Cytometry to evaluate S-phase entry (Fig. 7A). Cells were synchronized by serum withdrawal and then cells were released to the G_1_ phase by serum supplementation. At baseline, 0 h (before adding serum), the number of cells in G_1_ phase was not significantly different among the different plasmid-transfected cells (Fig. 7B). The fraction of cells in S phase, 16 h after serum supplementation, was 44.7% in control and 47.5% in THRA-expressing cells, but reduced to 37.5% (*p* < 0.01) in cells expressing THRA K283Q/K288R (Fig. 7C). The S phase lag persisted at 24 h in THRA K283Q/K288R-transfected cells, compared to control and THRA-transfected cells. The late S-phase entry delayed DNA synthesis and the expansion of cellular content, resulting in a reduced proliferation rate.

### Influence of the THRA K283Q/K288R on CREB- and AP1-mediated gene expression

**Figure 8:**
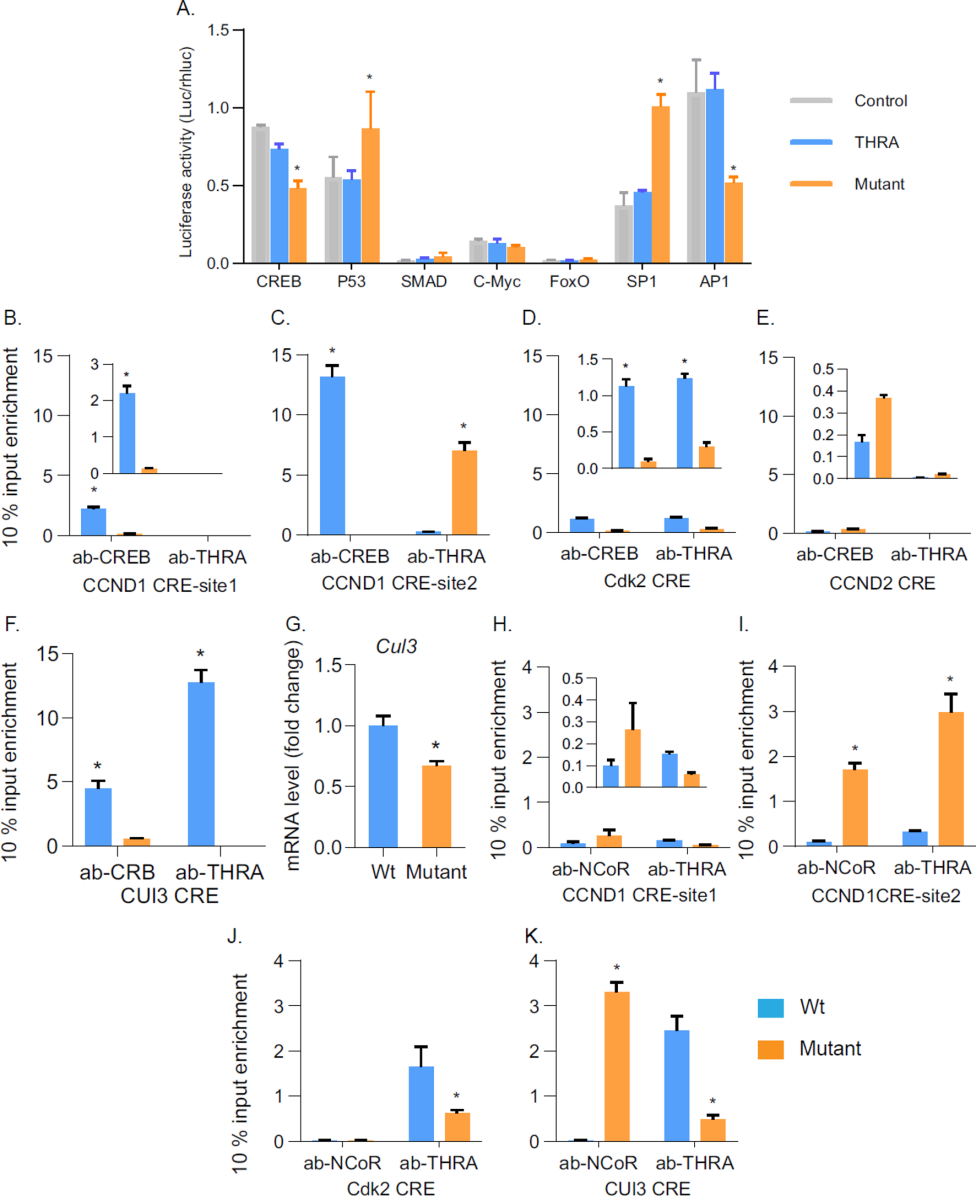
Expression of THRA K283Q/K288R associated with altered CREB binding to the cAMP Response Element (CRE) of cell cycle regulatory genes. (**A**) Human preadipocytes were transfected by electroporation with luciferase reporter containing multiple copies of a response element for each transcription factor tested. Each transfection had 6 replicates and the data shown is the average with SD. Statistical analysis was formed using One-way Anova. (**B–F**) ChIP analysis of CREB-THRA interaction on CREs. Preadipocytes were isolated from wild type mice and mutant mice. After synchronization, cells were supplied with serum for 8 h prior to harvest for ChIP assays of CRE on CCND1, CCND2 and Cdk4 promoter. For Cul3 gene promoter, cells were supplemented with serum for 12 h. (**G**) q-PCR analysis of *Cul3* gene expression in cells supplied with 12 h of serum after synchronization. (**H**–**K**) ChiP assay of THRA requirement of Nuclear Co-Repressor (NCoR) effects on CREs. The antibodies used were anti-THRA, anti-CREB, and anti-NCoR. PCR was performed for 35 cycles. Normal rabbit IgG was used as the negative control in ChIP assays. All ChIP data shown are after deducting the negative control and expressing as % input enrichment. Input was 10% of total pre-cleared lysate. Statistical analysis was performed using Student *t*-test. CREB, cAMP response element binding protein; *P53* tumor suppressor protein, *c-Myc* cellular homolog of myc oncogene,* FoxO1* forkhead box protein 1, *Sp1* specificity protein 1, *AP1* activator protein 1.

To determine in an unbiased fashion the signaling pathways disrupted by the desumoylated THRA, we screened the transcription factors important in the G_1_ phase regulation, using a functional reporter assay. CREB, P53, C-myc, SMAD, FoxO1, SP1 and AP1 are critical transcription factors for expression of genes important for cell cycle regulation^18–30^. CREB, C-myc, SP1 and AP1 are positive regulators of cell cycle progression^30–33^ and P53, SMAD and FoxO1 are negative regulators, promoting cell cycle exit^34–36^. We used luciferase reporters carrying multiple (6x) consensus response elements for each transcription factor and determined the functional activity of these transcription factors in cells transfected with empty vector (control), and vectors expressing THRA and THRA K283Q/K288R. The luciferase activity was not different between control and THRA-transfected cells for all transcription factor studied. However, in THRA K283Q/K288R-transfected cells, luciferase transcription-mediated by CREB and AP1 were reduced 46% (p < 0.05) and 53% (p < 0.05), respectively and P53 and SP1 were increased 31% (p < 0.05) and 53% (p < 0.05), respectively, compared to control cells (Fig. 8A). The transcription activity mediated by c-Myc, SMAD and FoxO1 was barely detectable. CREB, AP1 and SP1 are known to regulate the expression of *CCND1* (*cyclin D1*), *CCND2* (*cyclin D2*) and *Cdk2* and the response elements for these factors have been identified^19,22,27^. THRs has been previously shown to antagonize CREB-mediated transcription^37–39^. Based on these findings, we focused on the effects of the desumoylated THRA on CREB-mediated transcription for these key cell cycle genes.

### Altered THRA–CREB interaction in preadipocytes isolated from THRA K283Q/K288R mutant mice

THR does not bind directly to the CREB response element (CRE) but has been shown, in previous studies, to interact with CREB, as demonstrated by pull-down and co-IP assays^37,40,41^. We postulated that the sumoylation-defective THRA K283Q/K288R influenced protein–protein interactions between THRA and CREB, resulting in reduced expression of cell cycle genes. There are four potential CREs in the mouse *CCND1* gene, numbered *CCND1*-site1 to- site 4, equivalent to the human orthologue *CCND1* gene^32,42^. To detect the influence of THRA K283Q/K288R on protein-DNA and protein–protein interaction, we isolated preadipocytes from THRA K283Q/K288R mutant and wild type mice (abbreviated as mutant-preadipocytes and wt-preadipocytes). The mouse preadipocytes were synchronized by serum withdrawal for 36 h and then cultured with 10% serum for 8 h prior to harvesting nuclear protein for the ChIP assay. The initial ChIP assay, using anti-CREB antibody, showed that CREB bound to two of the four putative CREs (CCND1-site1 and CCND1-site2) in the G_1_ phase (Table1). In the remainder of the studies, only these two active sites were evaluated. In addition, the effects of THRA on CREB binding to CREs in the CCND2, Cdk2 and *Cullin3* (*Cul3*) gene promoters were investigated by ChIP assay (Table 1).

**Table 1:** Location of CREB binding sites in mouse gene and q-PCR primers for ChIP assays.

Gene	TF	Chr	TF-BS position (chr)	ChIP q-PCR length (bp)	ChIP detection	Primers for qPCR
CCND1	CREB	7	152123548	179	No	F: 152123639 5’-caccatgtctgtggtgaaaccact R: 152123460 5’-gtgcctctagaaatactaaggggac
CCND1	CREB	7	152124370	170	No	F: 152124490 5’-gatgtccactgagctcctgac R: 152124320 5’-gcgagacacaatggtggcgt
CCND1	CREB	7	152125045	165	Yes	F: 152125140 5’-tgcaggctgagcttctgtgg R: 152124975 5’-ataatattggcacgagcggcc
CCND1	CREB	7	152128311	170	Yes	F: 152128350 5’-gacctttcaagttgccgccag R: 152128209 5’-aggaaccctcgcgactaagc
Cdk2	CREB	10	128137056	107	Yes	F: 128137124 5’- gaggcagaggcagttggat R: 128137027 5’-ccagagagaatatatcggaacaa
Cdk2	CREB	10	128142068	139	No	F: 128142145 5’-ctatggaggtatagtcaggtat R: 128141946 5’-tctctgcctttctcagcctc
CCND2	CREB	6	127101069	143	Yes	F: 127101100 5’-gaggagatctaactgcccttcc R: 127100957 5’-aagagtggaaggtgggcgagc
CUL3	CREB	1	80337196	126	Yes	F:80337280 5’-tctgggccggatctcgtctc R:80337154 5’-gctccctttatcgcgctcct

CREB enrichment to the CCND1-site 2 was 7-times greater than that to CCND1-site 1 in wt-preadipocytes, indicating that the CCND1-site 2 is the primary CREB binding site (Fig. 8B,C). THRA from Wt-preadipocytes did not interact with CREB in either CCND1-site1 or site2 (Fig. 8B,C, orange bars). In mutant-preadipocytes, a very small amount of CREB was enriched to the CCND1-site1 and none to the CCND1-site 2. The THRA K283Q/K288R from mutant preadipocytes had significant enrichment (7.8%) to the CCND1-site 2 (Fig. 8C), indicating THRAK283Q/K288R is associated with reduced CREB DNA-binding.

The Cdk2 gene contains two potential CREs, but only one site had detectable CREB binding in our initial ChIP assays, which is referred as Ckd2 CRE (see Table 1 for location and sequence). In Wt-predipocytes, both THRA and CREB, were enriched at the Cdk2 CRE with similar enrichment, 1.3% and 1.2%, respectively (Fig. 8D). In mutant-preadipocytes, overall enrichment of CREB and THRA K283R/K288Q to this site was significantly reduced to 0.093% and 0.26% respectively. We speculate that fully sumoylated THRA facilitates CREB DNA binding on CDK2 CRE and THRA K283Q/K288R reduced CREB binding to the site. In the CCDN2 gene promoter region, low CREB enrichment was detected and THRA enrichment was negligible (Fig. 8E). Although the *CCND2* mRNA levels was reduced in mutant preadipocytes, it was not due to reduced activation by CREB but may be the result of reduced AP1. There is an AP1 binding site located in the *CCND2* promoter^23^. THRB is known to directly interact with AP1 protein^43,44^. The reporter assays demonstrated that AP1-mediated luciferase activity was reduced in mutant preadipocytes, consistent with reduced *CCND2* mRNA due to the influence of THRA K283Q/K288R on AP1-mediated transcription.

Cullin 3 (Cul3) is the core component of cullin-RING-based BCR (BTB-Cul3-RBX1) E3 ubiquitin-protein ligase complex, which targets cyclin E for ubiquitin-directed degradation and controls S-entry^45^. The S entry delay and reduced *Cul3* mRNA seen in human primary preadipocytes transfected with sumoylation-defective THRA K283Q/K288/R may be associated with altered CREB-mediated Cul3 gene transcription. We examined the CREB binding to CRE of the *Cul3* gene. In wt-preadipocytes, the enrichment of CREB and THRA to the CRE was 4% and 12%, respectively (Fig. 8F). THRA enrichment was greater than CREB, suggesting that THRA may be able to bind in the vicinity of the CRE and facilitate *Cul3* mRNA expression. In mutant-preadipocytes, the enrichment of THRA K283Q/K288R and CREB to the *Cul3* gene was eliminated, resulting in a reduction in Cul3 mRNA by 36% (*p* < 0.045) (Fig. 8G). The ChIP results indicate that in the presence of THRA K283Q/K288R CREB-mediated gene expression of *CCND1*, *Cdk2* and *Cul3*, was altered, consistent with the finding of delayed G_1_/S transition in THRA K283Q/K288R-transfected human preadipocytes.

### THRA K283Q/K288R is associated with reduced CREB binding to DNA and recruitment of NCoR

NCoR is a key regulator in THR-mediated transcription repression. NCoR protein contains two regions that specifically interact with unliganded THR^46^. Although THRA K283Q/K288R does not reduce T3 binding, as shown in a reporter assay, reduced sumoylation may alter the interface of THRA with other regulatory proteins. Using ChIP assays, we investigated whether an abnormal interaction between THRA K283Q/K288R and NCoR alters CREB-mediated transcriptions in Wt-preadipocytes. At the *CCND1*-site1, neither THRA nor NCoR were enriched (Fig. 8H**)**. At the *CCND1*-site2 (Fig. 8I), the enrichment of NCoR and THRA was detectable in very low levels in wt-preadipocytes, consistent with the lack of interaction between THRA and CREB on the *CCND1* gene (Fig. 8C). In contrast, in mutant-preadipocytes, both THRA K283Q/K288R and NCoR were highly enriched and CREB reduced on the CCND1-site2.

Enrichment of THRA, but not NCoR, was observed at the *Cdk2* CRE (Fig. 8J), indicating that THRA may interacts with CREB. At the *Cul3* CRE, NCoR enrichment was not detectable in Wt-preadipocytes, although THRA enrichment was dominant (Fig. 8K). In mutant-preadipocytes, NCoR was enriched at the *Cul3* CRE but not THRA K283Q/K288R, suggesting others factors contributing to NCoR enrichment. Concurrent recruitment of THRA K283Q/K288R and NCoR to *CCND1* CRE-site 2 suggests that desumoylation of THRA alters THRA interaction with NCoR and CREB.

The ChIP data suggests that reduced THRA sumoylation may play a role in THRA interaction with other transcription factors, such as CREB and could impact the expression of *CCND1*, *Cdk2* and *Cul3* genes. Additionally, THRA sumoylation may be important for interaction with NCoR.

## Discussion

TH is essential for cell proliferation during development and in regeneration after injury, as shown in a range of cellular and animal models^2^*.* TH regulates stem cell proliferation in the intestine, heart, brain, bone and skeletal muscle. In most tissues the stem cell proliferation is mediated by THRA, however, In the liver, regeneration of hepatic cells is regulated by THRB. THRA modulates the proliferation of the intestinal epithelial progenitors at the bottom of the crypts^3,47,48^. THRA/TH regulates cell growth and differentiation in the myocardium by stimulating specific genes and the AKT/mTOR pathway^49–51^. In the adult mouse brain, disruption of thyroid hormone signaling reduces neuroprogenitor survival, proliferation and differentiation^52–54^. In skeletal muscle THRA mediates myogenesis and tissue repair in response to injury^55^. THRA regulates bone growth and turnover via regulating differentiation of chondrocytes and osteoblasts and ossification^56,57^.

We used the THRA K283Q/K288R mouse model to investigate the impact of THRA sumoylation on adipocytes and fat deposition. In human primary preadipocytes, exogenous expression of mutant THRA (K283Q/K283R), was associated with reduced nuclear protein content of cyclin D1, cyclin D2 and Cdk2 and downregulation of gene expression, leading to the delay of G1/S transition. In our mouse model, mutation at the THRA-SUMO conjugation site, K283Q/K288R, was associated with reduced WAT stores and reduced proliferation of preadipocytes as assessed by in vivo and in vitro labeling studies.

In general, an increased fraction of adipose stem cells, preadipocytes, is characteristic of metabolically healthy fat with the ability to expand fat storage by hyperplasia and reduce fat deposition in organs^58^. Adipocyte diameter varies by fat depot as well as differences in fat depot characteristics between genders. Expansion of fat stores by adipocyte hypertrophy and larger adipocytes diameter has been associated with insulin resistance and metabolic diseases. A number of studies, however, have associated smaller adipocyte diameter with insulin resistance and Type 2 diabetes^59^. A clinical study with 40% overfeeding for 8 weeks showed that those with smaller adipocyte diameter at baseline had a greater increase in insulin resistance that those with larger adipocytes^60^. Single cell expression profiling of adipose progenitors has shown at least three distinct populations of pre-adipocytes, further showing the complexity of assessing the role of preadipocytes in expansion of adipocyte stores^61^. Our mice with a THRA sumoylation site mutation showed a reduction in fat stores and serum leptin, although did not show significant adverse metabolic consequences of reduced preadipocytes. Addition of a high fat diet resulted in a greater difference between mutant and wt mice in the fraction of body fat and fat stores. Reduced fat stores may also be the result of enhanced energy expenditure. The impact of the desumoylated THRA on thermogenesis and metabolic rate will need to be directly evaluated.

The THRA sumoylation mutant was associated with reduced preadipocyte proliferation in the in vitro and in vivo model, so we investigated the pathways that may be involved. In the cell cycle G0/G1 transition, D-type cyclins D1 and D2 are rapidly synthesized to meet the cellular proliferation demand. cAMP response element binding protein (CREB), is an important regulator of the cell cycle through direct regulation of genes, including *cyclin D1* (*CCND1), cyclin D2* (CCND2) and cyclin-dependent kinase 2 (*Cdk2)*^19,22,27^. THRs has been previously shown to antagonize CREB-mediated transcription by direct protein–protein interaction, independent of T3, as has been demonstrated previously in pituitary specific-Pit1 and interleukin (IL)-6 promoters^37–39^. A recent characterization of a desumoylated THRB showed a direct disruption of CREB-mediated gene transcription in the thyroid on genes important for thyroid hormone synthesis^15^. A functional screen of transcription response element promoters in our in vitro model, indicated reduced CREB signaling in the presence of desumoylated THRA.

SUMO conjugation alters receptor DNA-binding as well as interaction with binding partner and other transcription factors, altering gene expression and influencing cellular growth, proliferation and cellular function^62^. We utilized a ChIP assay to determine how the presence of the THRA sumoylation mutant influenced CREB signaling. The association of desumoylated THR with CREB inhibits CREB phosphorylation and ligand-dependent recruitment of coactivators, which impairs the CREB-mediated transcription and reduces cell proliferation^37,38^.

The role of THR/T3 in development has spatial and temporal specificity. THR may interact with various signals to facilitate the cellular demand in stem cells proliferation, although such activity does not occur in mature lineages. *CCND1*, *CCND2* and *Cdk2* mRNA level were reduced in G1 phase of mutant-preadipocytes. However, there was no change in mature adipocytes (WAT). The gene expression of D-type cyclins and Cdks are regulated by multiple transcription factors. For example, in addition to the two CREB binding sites, the *CCND1* promoter region, within 200 bp of transcription starting site (TSS) also contains binding sites for AP1, Ets, SP1, Egr1, TCF, NF_k_B and SATA5^63^. These transcription factors may act in spatial and temporal manner. CREB is involved in G1 phase regulation.

The mutation in THRA sumoylation site was associated with alterations in CREB binding, with an observed reduction in gene expression. The THRA mutant mouse model permitted us to study the physiologic function of THRA-SUMO conjugation, without disrupting the DNA binding and T3-mediated signaling properties of THRA. In a previous report, a mutation in the second zinc finger of THRB disrupted THRB augmentation of PI3K signaling, independent of T3, but did not impair direct TH action or THR binding to DNA^64^. THR residues that do not mediate direct nuclear signaling are important for modulating cell signaling pathways. A number of factors, independent of T3, have been shown to regulate cytoplasmic and nuclear localization of THR, including posttranslational modification by acetylation^65^. This nuclear-cytoplasmic shuttling may be importat in cell cylce regulation. THRA sumoylation may play a role in preadipocyte proliferation though CREB-mediated gene expression of G1 regulators.

The mouse with a THRA gene mutation and reduced sumoylation had smaller fat pads and adipocyte diameter, as well as a marked reduction in serum leptin. These differences in fat deposition between mutant and wt mice was increased by placing mice on a high fat diet. Alterations in metabolic rate may contribute to the reduced fat stores. We measured a reduction of preadipocyte proliferation in vitro and in an in vivo model. We hypothesize that reduced THRA sumoylation disrupted CREB signaling and interfered with cell cycle regulation. Although the role of preadipocytes likely varies by fat depot, a reduced fraction of preadipocytes are characteristic of obese individuals and those with insulin resistance. THR sumoylation may play a role in the regulation of preadipocyte proliferation and maintainance of cardiometabolic health.

## Materials and methods

### Animal use approval

Animal care and research protocol were approved by the institutional IACUC of VA Greater Los Angeles Healthcare System and in accordance with the Guide for the Care and Use of Laboratory Animals**.** All methods were carried out in accordance with ARRIVE Guidelines.

### Generation of sumoylation defective THRA K283Q/K288R mutant mice

The mutant mice were generated using FLP/FRT recombinase-mediated cassette exchange techniques (see Supplementary Fig. S1 online). The mutant mice were generated in C57BL/6NTac background. ES cells was derived from C57BL/NTac mice and manipulated to create the desired targeted mutation in the mouse *Thra* gene. The ES cells with the *Thra* gene mutation were used as donors and injected to the acceptor embryos of the C57BL/6NTac mice. The embryos were implanted back to C57BL/NTac female mice. The pups, then, have the exact C57BL/6NTac genome except the specific *Thra* mutation. The mutation is located at exon 8 of *THRA* gene. The mutations are located at 1367nt (A to C) and 1381 nt (A to G) based on NCBI accession # NM_178060.4. The homozygous mutation was confirmed by direct DNA sequencing and High Resolution Melting curve. The homozygous mice were then bred to the same strain for at least three generations prior to using them in experiments. We used in-house bred C57BL/6NTac mice with identical genetic background and living environment as the mutant mice for controls.

### Animal care and experiments

Mice were maintained in a standard 12-h light/12-h dark cycle with access to water and food ad libitum. Male mice were used in the study. Fasting serum T4, T3 and TSH and non-fasting serum leptin and adiponectin, were determined using Milliplex MAP magnetic Bead panel (Millipore Inc). Fasting serum cholesterol, free fatty acid and triglycerides levels were analyzed by Elisa Kit (Cayman Chemicals Inc). Fasting insulin using Elisa Kit from Crystal Chemical Inc.

### Western blot detection and quantification of THRA and sumoylated THRA

Subcutaneous fat was dissected from Wt and THRA K283Q/K288R mice (n = 3/genotype). The tissues were rinsed in PBS and immediately lysed in RIPA buffer with complete protease inhibitors and SUMO peptidase inhibitor 20 μM N-ethylmaleimide (final concentration). After centrifugation at 12,000×*g* for 10 min at 4 °C, the clear supernatant (total lysate) was collected. Two 10% SDS gels were prepared for western blots. Protein (35 μg/ lane) was loaded on the SDS gel. Upon completion of electrophoresis, the protein was transferred to PVDF membrane and Ponceau stained prior to blocking. The membrane #1 was blotted with anti-THRA (1:500, Abcam Inc) and #2 blotted with anti-SUMO2 (Developmental Studies Hybridoma Bank, 1:500). Membranes were imaged with Immobilon Forte Western HRP substrate (Millipore Inc). The western blots were quantified using Li-Cor Image Studio Lite. Western blot detected THRA and SUMO-THRA was normalized for total protein (Ponceau stained membrane) and presented as % total protein.

### Isolation and culture of mouse primary preadipocytes

Mouse primary preadipocytes were isolated from subcutaneous adipose tissue of wild type and THRA K283Q/K288R mutant mice using the method described previously^66^. In brief, 6 week-old male mice (n = 6) were euthanized. Subcutaneous fat pads were dissected, washed with PBS, and minced in 1 mL of advance DMEM/-F12 medium containing dipeptidase II (2.4 U/mL/10 mM CaCl_2_) and collaginase D (1.5 U/mL). After 1 h digestion at 37 °C with agitation, the digestion was stopped by adding complete DMEM medium containing 10% FBS. After centrifuge for 10 min at 700 × g, the pellet (stromal vascular fraction) was collected, resuspended in 1 mL PBS, and filtered through a 40 μM cell strainer, and then plated to 1% collagen coated plates. After 1 h of plating, cells were carefully washed with warm PBS to remove unattached debris and fresh medium was added. The isolated cells were used immediately in subsequent experiments.

### Chromatin immunoprecipitation (ChIP) assay

Preadipocytes isolated from inguinal fat were synchronized to G_1_ phase as described the above. After the cells were re-fed with 10% calf serum for 8 h, cells were fixed with formaldehyde (final concentration 1%) for 10 min. Fixation was then stopped with the addition of glycine. The remainder of the procedure followed the manufacturer’s instructions for ChIP Kit (Qiagen Inc). For each ChIP reaction, 2 million cells were used. The antibodies used were anti-THRA (ab2743 ChIP grade, rabbit, Abcam Inc.), anti-NCoR (17-10260, ChIP grade, rabbit, Millipore Inc.), and anti-CREB (ab31387, ChIP grade, rabbit, Abcam Inc.). Normal rabbit IgG was used as negative control. The Input was 10% pre-cleared total lysate. The purified DNA was PCR amplified for 35 cycles with triplicates. The data shown was deducted from negative control and expressed as % Input enrichment. ChIP q-PCR primers were Qiagen pre-designed and are shown (Table 1).

### In vivo EdU (5-ethynyl-2’-deoxyuridine) labeling of dividing preadipocytes in mice

EdU (Invitrogen) was prepared 50 μg/mL in saline and was administered to 3 week old mice (n = 2) at 50 μg/g BW (i.p.). Twelve hours after injection, mice were euthanized and inguinal fat was fixed in 4% paraformaldehyde in 4 °C overnight. The frozen tissue blocks were sectioned (12 μM thickness). The EdU was detected by Click-IT chemistry following the manufacturer instructions (Thermofisher Scientific Inc.). The sections were then washed with PBS and blocked in blocking buffer (10% goat serum and 0.03%tritonX-100 in PBS) for 30 min prior to incubation with anti-β-catenin antibody (Santa Cruz Biotech.) at dilution 1:25 overnight at 4 °C, followed by goat-anti-rabbit Alexa Fluro 568 at dilution 1:2000 for 2 h at room temperature. The section was mounted with a coverslip using ProLong Gold anti-fade reagent containing DAPI for staining nucleus. The sections were searched at × 10 magnification for vascular stromal areas and then these areas were examined in a × 20 magnification field. EdU-incoporated dividing cells were quantified by counting the green fluorescent labeled nucleus in three consecutive fields with 20X magnification in each mouse.

### EdU labeling of proliferating preadipocytes cells isolated from mutant mice

Cells were isolated from the subcutaneous WAT of 6 week old wild type and mutant mice, grown to contact inhibition (48 h post 100% confluence), and then re-plated to 4-chamber slides at 2 × 10^4^ cells per chamber. EDU (Clik-it EDU kit) was added to culture medium at a final concentration of 10 μM and incubated for 4 h in the culture incubator. Following fixation, cells were stained with Alexa Flour (594 or 488) for imaging. The number of EdU incorporated cells were quantified by counting total visible cells in a slide for 6 randomly selected frames and divided by the total visible DAPI labeled cells in these frames and presented as % DAPI stained cells.

### Cell diameter quantification

Mice (n = 3/group) were fed with regular chow diet. The tissues were fixed by trans-cardial perfusion with 4% paraformaldehyde. Subcutaneous WAT was collected and paraffin embedded to prepare sections. The histology sections were imaged using Apical Imager. The cell diameter was quantified using ImageScope at similar location of inguinal fat pads of Wt and mutant mice. For each genotype, three sections were quantified at maximum enlargement for three consecutive frames. A total of 360 cells were measured for each genotype.

### Quantification of total number of preadipocyte isolated from subcutaneous WAT of each mice

Preadipocytes were isolated from stromal vascular fraction (SVF) of subcutaneous fat of each mice (n = 3/genotype) as described above. Cells were collected and diluted in DMEM at low density. The number of preadipocytes were counted, using a hemocytometer, for 6 aliquots from each mouse.

### Cell culture and transfections

Human primary preadipocytes from non-diabetic donors (Lonza Inc) were maintained in PBM-2 medium with 10% FBS supplement (Lonza Inc). Cells were transfected with control plasmid (empty vector) and plasmids expressing hTHRA or hTHRA K283Q/K288R by electroporation, using a 4D Nucleofector device and primary cell-specific reagent (Lonza Inc).

### G_1_ phase synchronization

Primary human preadipocytes (Lonza Inc) were grown to complete confluence and maintained for 48 h post-confluence. Cells were then trypsinized, washed twice with warm PBS and cultured in PBM-2 medium without serum for 48 h. Primary preadiocytes isolated from the inguinal fat of Wt and THRA K283Q/K288R mutant mice were grown in DMEM with 10% calf serum to 80% confluence, then cultured for 3 days in DMEM with 0.5% calf serum before serum withdrawn. We observed that human primary preadipocytes tolerated 72 h of serum withdrawal without signs of apoptosis, but primary preadipocytes isolated from mice tolerated only 36 h of serum withdrawal. Preadipocytes from mice were, therefore, serum starved for 36 h to achieve G_1_ synchronization. The number of cells synchronized to G_0_/G_1_ was measured by flow cytometry. To release cells to the cell cycle, 10% serum was supplied for the period (0, 8, 16 and 24 h). Cells that underwent prolonged serum withdrawal required longer than the usual period in G_1_ phase to recover the cellular content and cellular activity before entering S phase. For this reason, G_1_/S transition was analyzed 9 h after the cells were refed with serum.

### Immunofluorescent (IF) staining and confocal imaging

Human primary preadipocytes were plated in chamber slides. Cell culture conditions, serum withdrawal and refeeding conditions, were the same as described for cell synchronization studies. Cells, 9 h after serum refeeding, were fixed with 4% paraformaldehyde, permeabilized in 0.2% TritonX-100/PBS, and then blocked with 5% goat serum in PBS. Cells were incubated with primary antibodies at 1:100 concentration overnight and secondary antibodies at 1: 250 for 1 h. Cells were then washed, mounted with Prolong Gold/DAPI (Life Science Inc.) and imaged using Zeiss Laser Scanning Microscope (LSM 710). The details on the antibodies details, see the section describing Western blot and IP/Co-IP procedures.

### Cell cycle analysis by flow cytometry

Human primary preadipocytes were synchronized by serum withdrawal for 48 h. Cells were collected at various time points (0, 8, 16 and 24 h), after supplementation with 10% serum, and fixed for flow cytometry analysis with propidium iodine.

### Quantitative RT-PCR analysis

Total RNA was isolated from cells (Qiagen kit). Primers used in q-PCR were pre-evaluated by Qiagen. The data were normalized with four housekeeping genes (GAPDH, beta-Actin, beta2-microglobulin and HPRT) and shown as the average of triplicate determinations.

### RNA-seq analysis of gene expression

Total RNA was isolated from Inguinal fat of 3 month old male mice (n = 3/genotype) and used in RNA-seq analysis. The library construction, RNA-seq and data analysis were performed by the Genomic Core Facility at UCLA.

### Fractionation of nuclear and cytoplasmic proteins, immunoprecipitation (IP/co-IP) and Western Blot

Nuclear and cytoplasmic proteins were isolated using a Nuclear and Cytoplasmic Extraction Reagent (NE-PER) Kit (ThermoFisher Inc.) following the manufacturer’s instructions. The fractioned components were used for IP/co-IP experiments. After IP/Co-IP, protein was separated by 10% SDS-PAGE, blotted to a PVC membrane, detected with antibodies, imaged, and quantified using Li-Cor Image Studio Lite. Antibodies used for IP/co-IP/WB were anti-cyclin D1 (sc-20044, mouse), anti-cyclin D2 (sc-181, rabbit), anti-CDK4 (sc-260, rabbit) and anti-Cdk2 (sc-163, rabbit), obtained from Santa Cruz Biotech Inc. Antibodies for p27^kip^ (3686, rabbit), were from Cell Signaling Tech. All antibodies were used at 1:500 dilution for Western blot and 1:50 dilution for IP and co-IP experiments.

### Luciferase reporter assay

Human primary preadipocytes were grown to contact inhibition, so cells would cease to proliferate. Cells were then trypsinized and transfected with Luciferase reporter using 4D Nucleofector device and plated to 96-well dishes at 60% confluence, allowing cells to proliferate. Each Luciferase reporter (Qiagen Inc) contains six copies of a consensus response element for the transcription factors tested. Luciferase activity was determined 10 h after transfection using a Dual luciferase kit (Promega Inc.). The data are shown as the mean value of 6 replicates with standard deviation (SD).

### Statistical analysis

PCR data was analyzed by 2-tailed student *t*-Test for paired comparison. For other experiments, One-way ANOVA or two-way ANOVA were used for statistical analysis. All data represent the mean value with standard deviation (SD). *P* ≤ 0.05 was considered statistically significant.

## Supplementary Information

Supplementary Information.

## Data Availability

All data generated or analyzed during this study are included in this published article (and its Supplementary Information files). RNAseq data has been deposited in NCBI Gene Expression Omnibus (GEO) access GSE182723.
