# Supplementary material for: Thyroid hormone receptor alpha sumoylation modulates white adipose tissue stores
Source: Sci Rep. 2021 Dec 16;11:24105. doi: 10.1038/s41598-021-03491-6 (PMC8677787; doi:10.1038/s41598-021-03491-6)
Supplement: Supplementary file 1 — Supplementary Information. [file 41598_2021_3491_MOESM1_ESM.docx]

**Thyroid Hormone Receptor Alpha Sumoylation Modulates White Adipose Tissue Stores**

Yan-Yun Liu^1,*^, Jingjing Jiang^1,a^, Sujie Ke^1,b^, Anna Milanesi^1^, Kiyomi Abe^1,c^, Gilberto Gastelum^1^, Jianrong Li^1,b^ Gregory A. Brent^1,*^

^1^Division of Endocrinology, Diabetes and Metabolism, Departments of Medicine and Physiology, David Geffen School of Medicine at UCLA, and Veterans Affairs Greater Los Angeles Healthcare System, Los Angeles, CA 90073

^a^ Present address: Department of Endocrinology, Zhongshan Hospital, Fudan University, Shanghai 200025, China.

^b^ Present address: Department of Endocrinology, Union Hospital, Fujian Medical University, Fuzhou 350001, China.

^c^ Present address: Department of Pediatrics, Keio University School of Medicine, Tokyo 160-8582, and Saiseikai Central Hospital, Tokyo 108-0073, Japan.

***, Corresponding authors**: Yan-Yun Liu and Gregory A. Brent

**Email:**  YYL ([yyl@g.ucla.edu](mailto:yyl@g.ucla.edu)) or GAB ([gbrent@mednet.ucla.edu](mailto:gbrent@mednet.ucla.edu)).


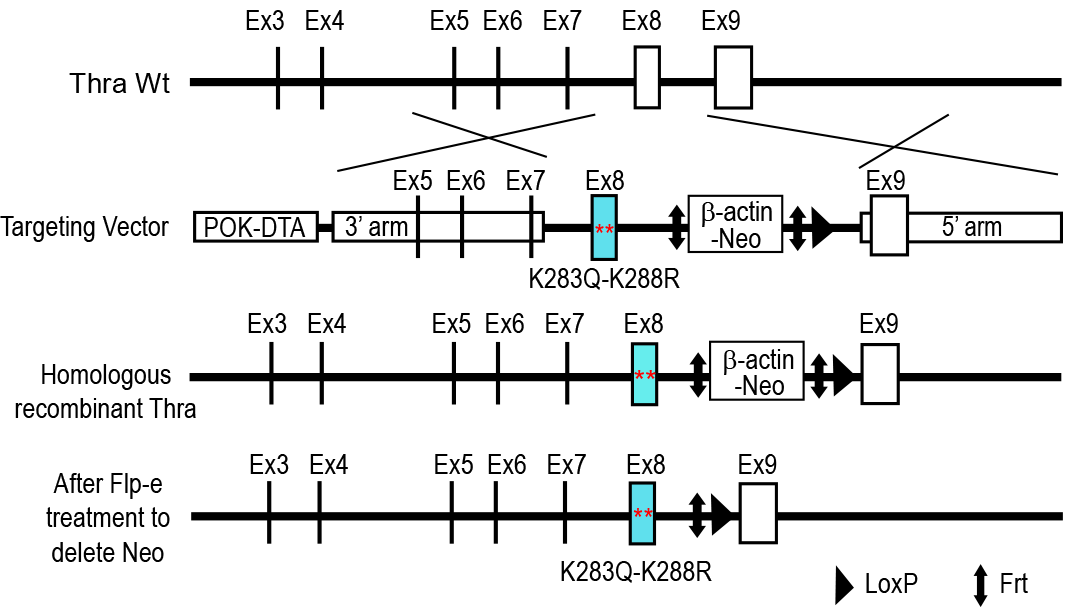


**Supplemental Figure 1.** Generation of a “Knock-In” allele to mutate the *Thra* gene by a sequence replacement strategy. The targeting construct contains the K283Q and K288R mutations in exon 8, a 5.2kb 5’ arm of homology (containing exons 5-7), a 5.2 kb 3’ arm of homology (containing exon 9), a Diphtheria Toxin A (DTA) cassette, and a Neomycin (Neo) cassette flanked by frt sites for selective deletion. The Neo element allows for positive selection in Embryonic Stem (ES) cells, while the DTA element permits negative selection. After homologous recombination of the “Knock-In” construct, the Neo is excised *via* Flp-e administration. The resulting homologous recombinant THRA allele contains the K283Q and K288R mutations.


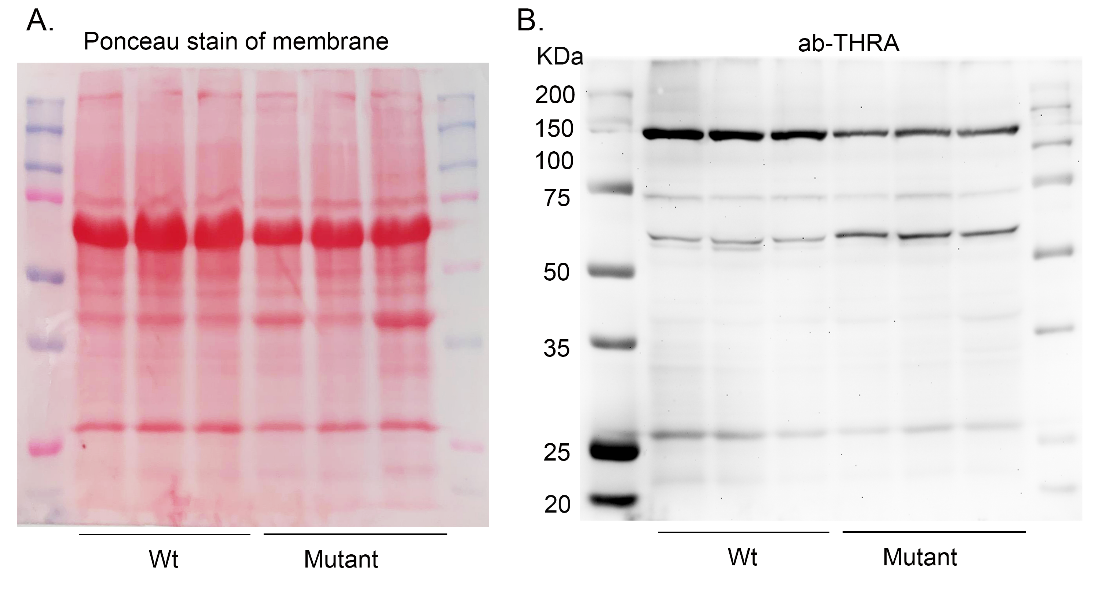


**Supplemental Figure 2.** Western blot detection of THRA and sumoylated THRA. Subcutaneous fat was dissected from Wt and THRA K283Q/K288R mice (n=3/genotype). The tissue was lysed in RIPA buffer with complete protease inhibitors and 20 μM N-ethylmaleimide, a SUMO peptidase inhibitor. Two identical 10% SDS gels were prepared for western blot analysis. Protein (35 μg/ lane) was loaded on the SDS gel. After completion of electrophoresis, the protein was transferred to a PVDF membrane. (A) Membrane stained with Ponceau stain prior to blocking. (B) Membrane blotted with anti-THRA Ab (1:500, Abcam Inc).


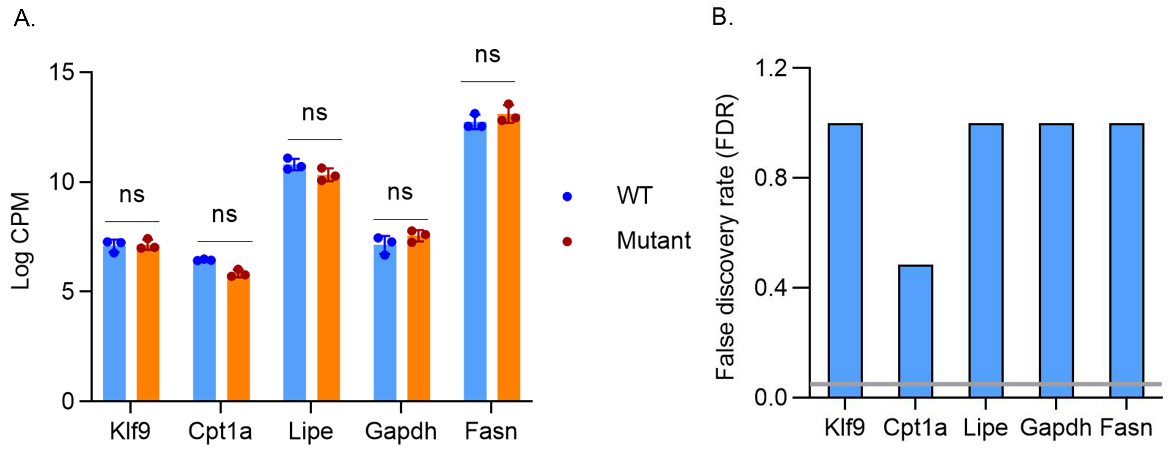


**Supplemental Figure 3.** Gene expression of thyroid-hormone dependent genes and controls in white adipose tissue.

Mice (n=3/genotype), 3 months old, were euthanized and inguinal fat was isolated. RNA was extracted and RNA-seq analysis performed. The magnitude of mRNA expression is shown as Log CPM (A) and the expression significance was determined by False Discovery Rate (FDR) (B) All comparisons of mRNA expression comparing wild-type (wt) and mutant mice were not significant (ns, FDR >0.05). Klf9-Kruppel Like Factor 9, Cpt1a-Carnitine Palmitoyltransferase 1A, Lipe- Hormone Sensitive Lipase, Gapdh-Glyceraldehyde 3-phosphate dehydrogenase (control), Fasn-Fatty Acid Synthase


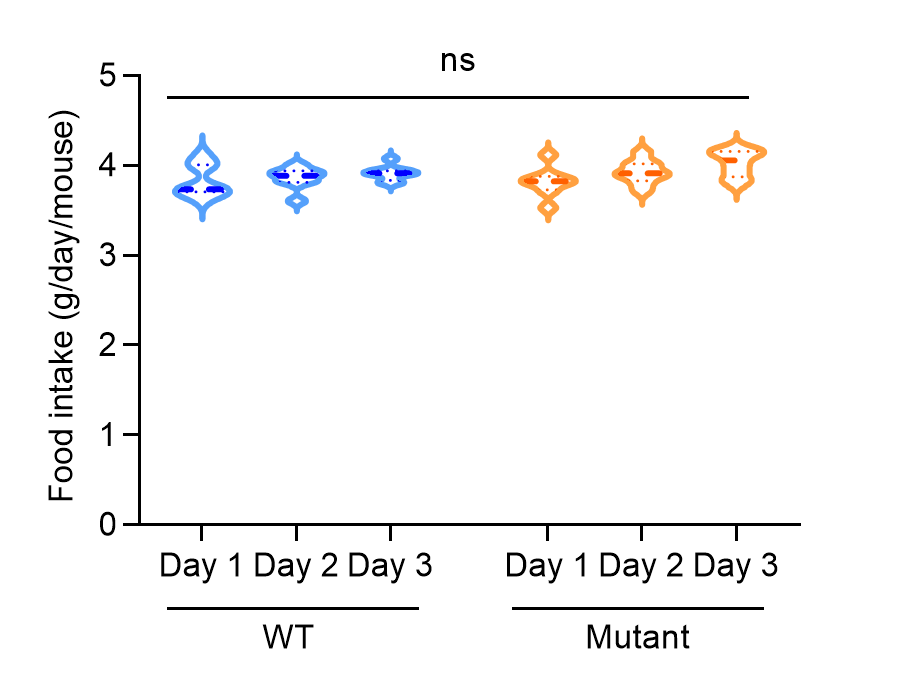


**Supplemental Figure 4.** Food intake in wild-type (Wt) and THRA K283Q/K288R mice. Three month old male mice (n=8/genotype) were placed in metabolic cages with free access to food and water and acclimated to the cages for one week. Data for daily food intake was acquired for three consecutive days. The data is presented as average daily food intake (g/day/mouse) as well as the distribution of individual values. Statistical analysis was performed using One-Way ANOVA. ns-not significant


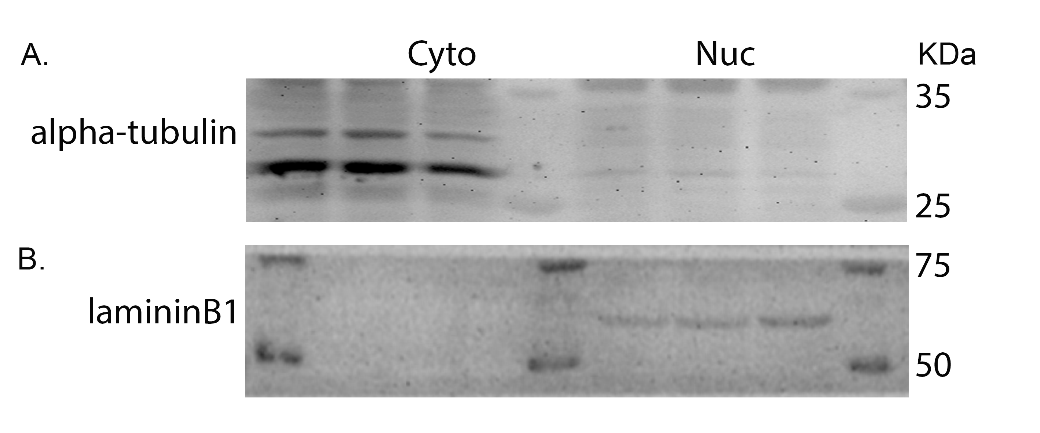


**Supplemental Figure 5. Markers for cytoplasmic and nuclear fractions.**

Human preadipocytes were serum starved for 48 hours and released into cell cycle by addition of 10% serum to the culture media. After 9 hours, cells were collected and fractionated into cytoplasmic (Cyto) and nuclear (Nuc) fractions. The quality of fractionation was analyzed by immunoblots with (A) anti-alpha tubulin Ab as a cytoplasmic marker (1:500) and (B) anti-laminB1 Ab as a nuclear fraction marker (1:500). Both antibodies were from Santa Cruz Biotechnology. The blots (panels A and B) were cut prior to hybridization and the origin blots are provided (see Supplementary Fig. S10 online).


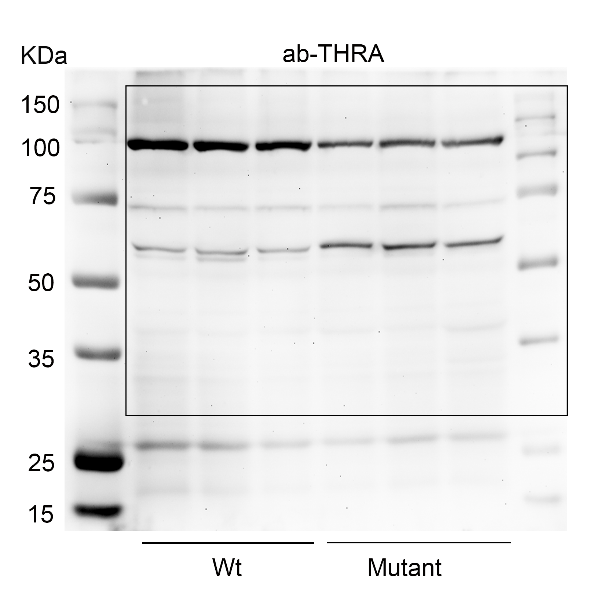


**Supplemental Figure 6. Full Western blot image for figure 1C.** Framed region indicates the cropped portion used in Figure 1C. This full Western blot is the same blot shown in supplementary figure 2B.

A.


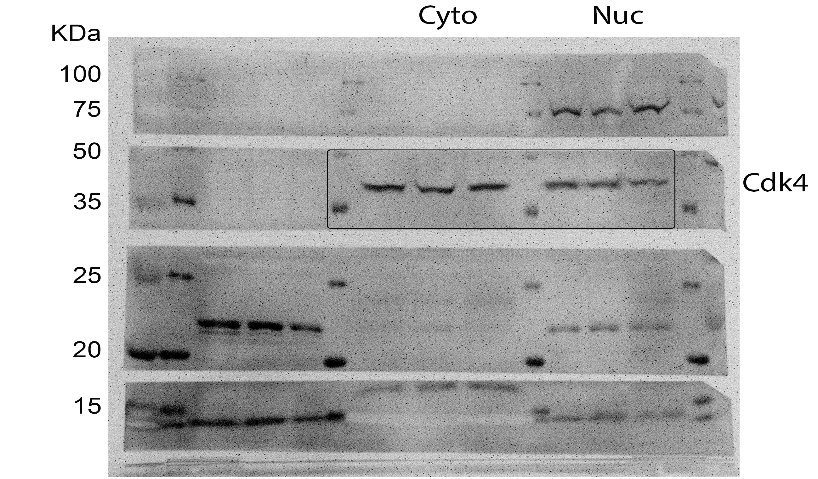


B.


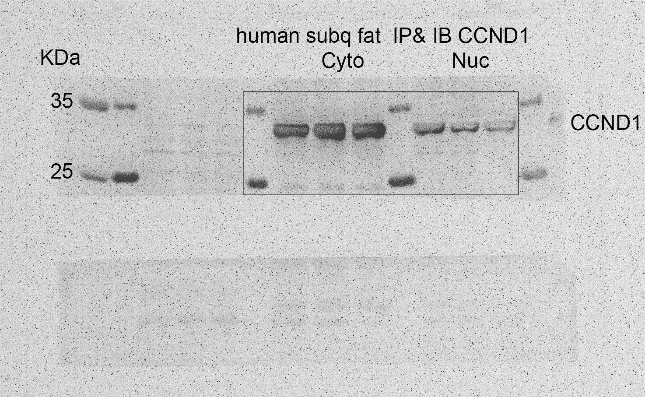


C.


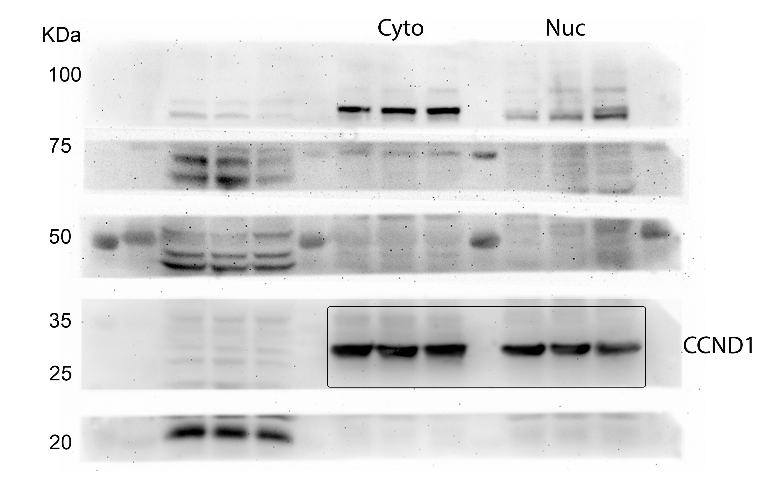


**Supplementary Figure 7.** Full image of origin immunoblots. The framed regions were used in Figure 5B and 5C. (A) IP-IB membrane with anti-Cdk4 Ab for Figure 5B and (B) IP-IB blot with anti-CCND1 Ab for Figure 5C. (C) CCND1 Western blot with anti-CCND1 antibody. A complete membrane that includes CCND1 detection from a similar experiment is shows to indicate the overall position of the positive CCND1 band. The membranes were divided horizontally in order to detect multiple proteins with different antibodies. The membrane fragments are aligned sequentially to show the origin blot. Cyto-cytoplasmic, Nuc-nuclear

A.


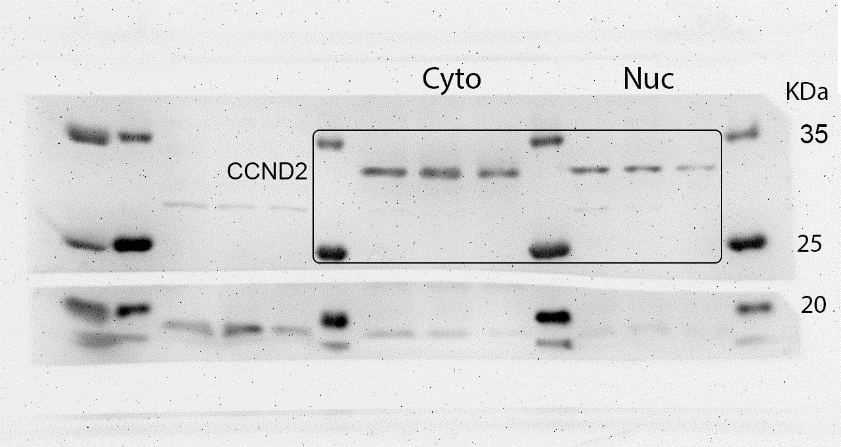


B.

**
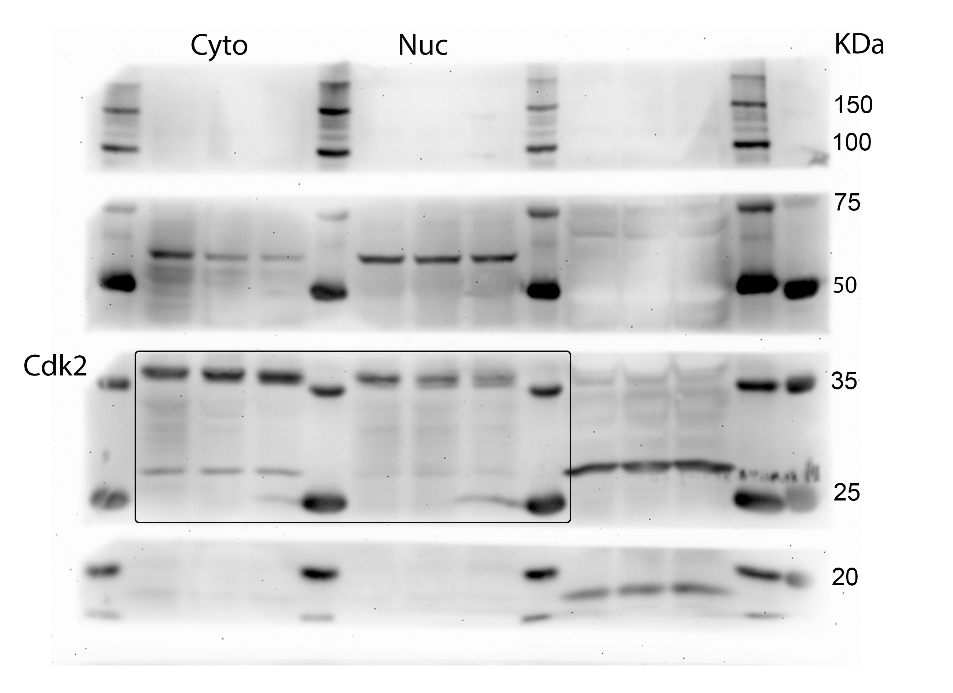
**

**Supplementary Figure 8. Full images of blots.** (A) IP-IB blot with box showing the anti-CCND2 section used in Figure 6B (B) The IP-IB blot with box showing the anti-Cdk2 used in Figure 6F. The membranes were divided horizontally in order to detect multiple proteins with different antibodies. The membrane fragments are aligned sequentially to show the origin blot.

A.

**
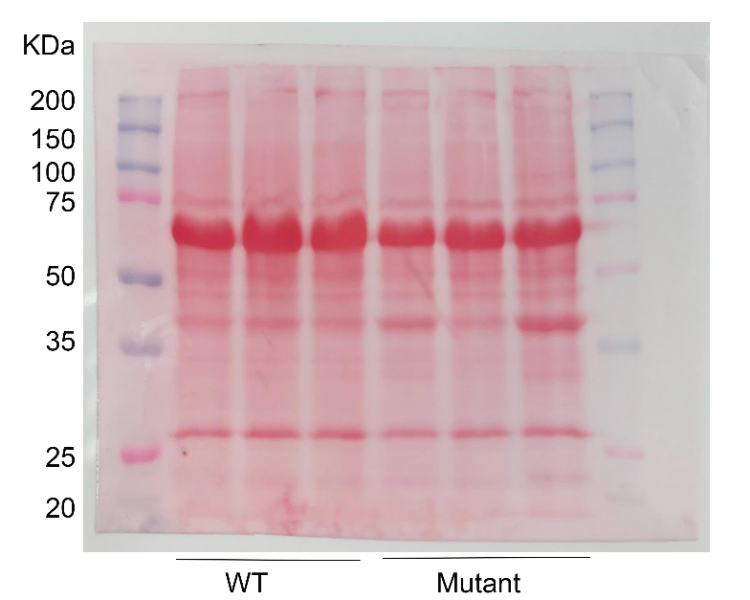
**

B.

**
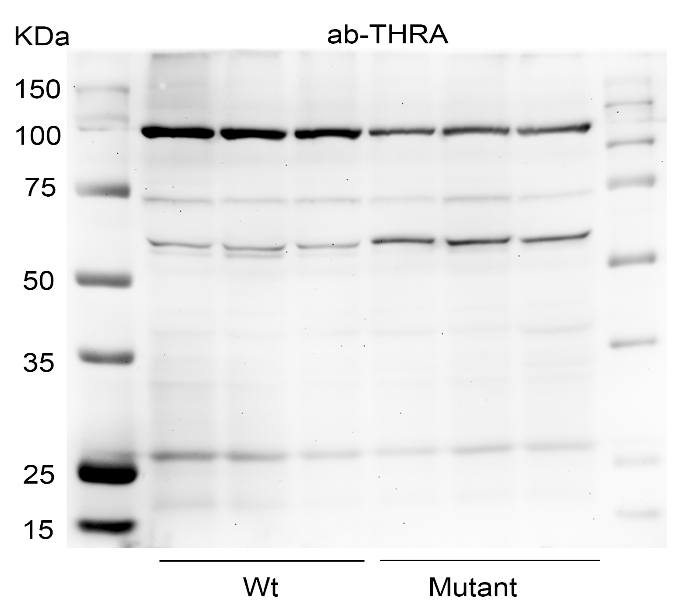
**

**Figure 9. Images of membranes used in Supplementary Figure 2.** (A) Ponceau red stain of membrane used in S Figure 2A and (B) Western blot with anti-THRA Ab used in Supplementary Figure 2B.

A.


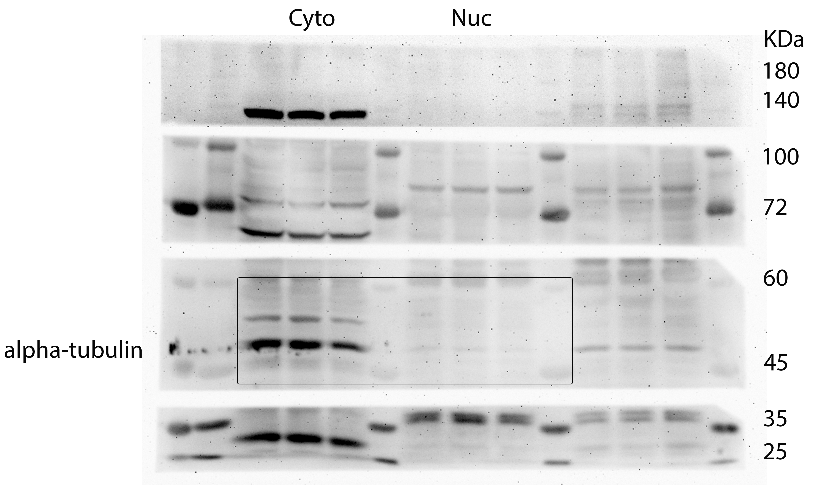


B.


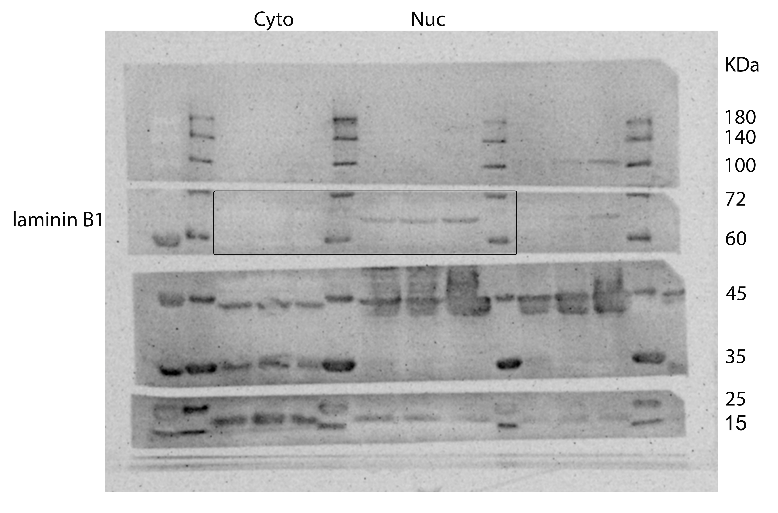


**Figure 10. Western blot images for Supplemental Fig 5.** (A) Western blot with anti-alpha tubulin for S Fig 5A and (B) Western blot with anti-lamin B1 used in S Fig 5B. The membranes were divided horizontally in order to detect multiple proteins with different antibodies. The membrane fragments are aligned sequentially to show the origin blot. Cyto-cytoplasmic, Nuc-Nuclear

**Supplemental Table 1.** RNA-seq IPA (QIAGEN software) analysis of canonical pathways (Z-score >0.5)

RNA was from 3 months old mice (n=3/genotype).

| **Ingenuity Canonical Pathways** | **-log(p-value)** | **z-score** | **Molecules** |
| --- | --- | --- | --- |
| Actin Cytoskeleton Signaling | 5.31 | 3.317 | ACTC1,ACTN2,FGF13,KNG1,MYH1,MYH2,MYH3,MYH7,MYH7B,MYH8,  MYL2,MYL6B,PAK3 |
| ILK Signaling | 5.2 | 2.309 | ACTC1,ACTN2,FLNC,KRT18,MYH1,MYH2,MYH3,MYH7,MYH7B,MYH8,MYL2,MYL6B |
| Protein Kinase A Signaling | 1.86 | -0.816 | AKAP6,FLNC,MYH2,MYL2,MYL6B,PDE6H,PPP1R3C,PTPN3,PTPRQ,  TNNI1,TULP2 |
| RhoGDI Signaling | 1.55 | -2.236 | ACTC1,CDH3,GNAL,MYL2,MYL6B,PAK3 |
| Regulation of Actin-based Motility by Rho | 1.49 | 2 | ACTC1,MYL2,MYL6B,PAK3 |
| CXCR4 Signaling | 1.2 | 2 | EGR1,GNAL,MYL2,MYL6B,PAK3 |
| LXR/RXR Activation | 1.2 | 2 | AGT,ALB,APOD,KNG1 |
| RhoA Signaling | 1.14 | 2 | ACTC1,LPAR4,MYL2,MYL6B |
| Signaling by Rho Family GTPases | 1.03 | 2.23 | ACTC1,CDH3,GNAL,MYL2,MYL6B,PAK3 |
| Leukocyte Extravasation Signaling | 0.97 | 0.5 | ACTC1,ACTN2,MMP12,MMP24,  THY1 |
| Integrin Signaling | 0.86 | 1.34 | ACTC1,ACTN2,ITGAD,MYL2,PAK3 |
| cAMP-mediated signaling | 0.777 | 1.12 | AKAP6,GNAL,GPR17,PDE6H,  TULP2 |
| Cardiac Hypertrophy Signaling | 0.74 | 1 | ADRA1D,GNAL,MAP3K15,MYL2,  MYL6B |
| Production of Nitric Oxide and Reactive Oxygen Species in Macrophages | 0.74 | 1 | ALB,APOD,MAP3K15,PPP1R3C |
| Cardiac Hypertrophy Signaling (Enhanced) | 0.66 | 2 | ADRA1D,AGT,FGF13,HSPB7,MAP3K15,PDE6H,TULP2,WNT2B |
| Colorectal Cancer Metastasis Signaling | 0.394 | 1 | MMP12,MMP24,PTGER1,WNT2B |
| Sirtuin Signaling Pathway | 0.292 | 1 | ARNTL,MT-ND6,TUBA1A,UCP2 |

**Supplemental Table 2.** IPA (QIAGEN software) analysis of Upstream regulator

(Top 10 upstream regulators)

| **Upstream Regulator** | **Expr Fold Change** | **Activation Z-score** | **p-value of overlap** | **Targeted molecules in dataset** |
| --- | --- | --- | --- | --- |
| PER | -2.7 | -1 | 7.49E-07 | ARNTL,DBP,PER2,PER3,UCP1 |
| UCP1 | -3.76 | -1.41 | 0.0000752 | ANKRD1,CCDC136,CTH,MSS51,MYH1,  MYH2,SLN,SMOX,UCP1,UCP2 |
| AGT | 2.31 | 1.43 | 0.00173 | ACAT2,AGT,ATP1B1,CDKN1A,EGR1,ERRFI1,FGF13,GSTA3,MYH7,NOS1,PPP1R3C,REN,SCN2A,SLC5A2,UCP2 |
| EPCAM | -6.29 | -1.53 | 0.00302 | CDKN1A,EGR1,EPCAM |
| NPAS2 | 4.388 | 1.22 | 0.00358 | ARNTL,PER2 |
| ARNTL | 3.03 | -1.09 | 0.00487 | ARNTL,DBP,PER2,PER3 |
| ISL1 | 6.21 | 0.152 | 0.029 | ACTC1,BNC1,CALB2,FGF13 |
| WT1 | 66.659 | 1.619 | 0.003 | CDKN1A,EGR1,FOSB,MSLN,NRA,  SLC15A2,WT1 |
| REN | 100.66 | 1.13 | 0.0066 | AGT,MYH7,REN |
